# Quantifying the mutational landscape of retroviral and lentiviral vectors in gene therapy patients

**DOI:** 10.1016/j.ymthe.2025.05.034

**Published:** 2025-05-30

**Authors:** Kevyn L. Hart, Ralph Valentine Crisostomo, Annika Mittelhauser, Lingyu Zhan, Nika Kononov, Kathryn Bradford, Donald B. Kohn

**Affiliations:** 1 Department of Human Genetics, David Geffen School of Medicine, University of California, Los Angeles, Los Angeles, CA 90095, USA; 2 Molecular Biology Interdepartmental Program, David Geffen School of Medicine, University of California, Los Angeles, Los Angeles, CA 90095, USA; 3 Department of Microbiology, Immunology & Molecular Genetics, David Geffen School of Medicine at University of California, Los Angeles, Los Angeles, CA 90095, USA; 4 Department of Psychiatry, Center for Neurobehavioral Genetics, Semel Institute for Neuroscience and Human Behavior, University of California, Los Angeles, Los Angeles, CA 90095, USA; 5 The Collaboratory, Institute for Quantitative and Computational Biosciences, University of California, Los Angeles, Los Angeles, CA 90095, USA; 6 Department of Pediatrics, David Geffen School of Medicine at University of California, Los Angeles, Los Angeles, CA 90095, USA; 7 Department of Molecular and Medical Pharmacology, David Geffen School of Medicine at University of California, Los Angeles, Los Angeles, CA 90095, USA; 8 The Eli & Edythe Broad Center of Regenerative Medicine & Stem Cell Research, University of California, Los Angeles, Los Angeles, CA 90095, USA

**Keywords:** ADA-SCID, APOBEC3, reverse transcriptase, gene therapy, lentiviral vectors

## Abstract

Adenosine deaminase severe combined immunodeficiency (ADA-SCID) is a monogenic disorder caused by mutations in the *ADA* gene. Gene therapy using γ-retroviral and lentiviral vector gene addition approaches have shown curative results. We sequenced the *ADA* transgene in transduced CD3^+^ T cells, and in peripheral blood cells from patients treated with autologous CD34^+^ cells transduced with either a γ-retroviral or lentiviral *ADA* gene vector to assess transgene mutational profiles. In both CD3^+^ T cells and ADA-SCID patients’ cells treated with the lentiviral vector, we observed significantly higher occurrences of guanine (G)-to-adenosine (A) base substitutions than with the γ-retroviral vector. We hypothesized that this G-to-A mutational signature was due to the APOBEC3 cytosine deaminase protein family. By knocking out APOBEC3 genes in HEK239T packaging cells, APOBEC3-mediated mutagenesis decreased by 91.2% along the transgene in CD34^+^ transduced cells in comparison to CD34^+^ cells transduced with lentiviral supernatant packaged in parental HEK293T cells.

## Graphical abstract

**Figure:**
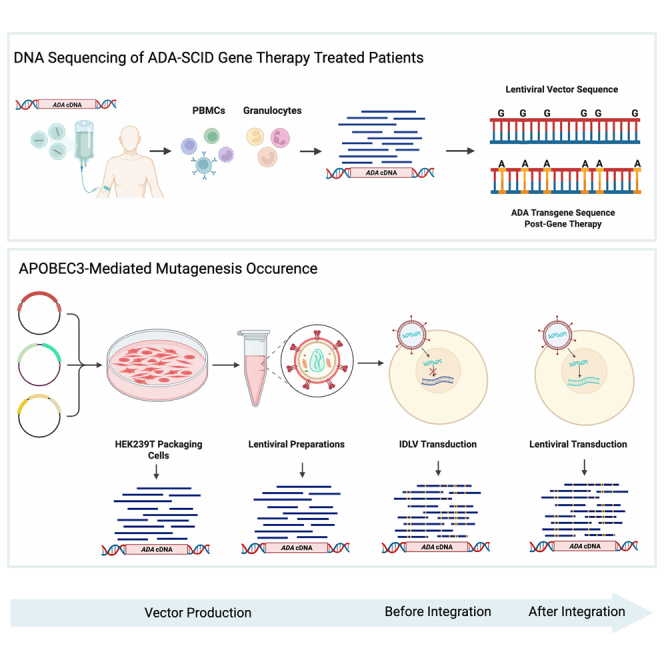

## Teaser

DNA sequencing of ADA-SCID patients’ cells treated with a lentiviral vector revealed a high frequency of G-to-A base substitutions across the integrated transgene, driven by APOBEC3 cytosine deaminases. Knocking out APOBEC3 genes in HEK293T packaging cells significantly reduced this mutagenesis, identifying APOBEC3 activity as a significant factor influencing transgene fidelity.

## Introduction

Adenosine deaminase severe combined immunodeficiency (ADA-SCID) is a monogenic disorder caused by mutations in the *ADA* gene of purine metabolism. ADA-SCID is a life-threatening condition because it results in severely impaired immune function. Specifically, ADA deficiency prevents the development of lymphocytes that are critical in protective adaptive immune responses. Enzyme replacement therapy with ADA is a long-term treatment that requires repeated weekly doses throughout the patient’s life. Allogeneic hematopoietic stem cell transplantation is a curative option but has the best results with an human leukocyte antigen-type matched sibling, to minimize the risk of graft-versus-host disease.

Gene therapy first became a course of action for the treatment of inherited monogenic diseases with the development of engineered retroviruses. Retroviral vectors have the ability to deliver an exogenous transgene into patients’ cells by converting their RNA genome into double-stranded DNA with reverse transcriptase (RT) and integrating that DNA into the host’s genome.^1^ The first type of viral vector utilized a gamma-retrovirus, murine leukemia virus (MLV), to successfully deliver the therapeutic transgene into the target cell. It has been nearly 35 years since the first patient received viral vector gene therapy in 1990 for the inborn error of immunity ADA-SCID.^2^ The initial results proved that retroviruses can be developed as a gene transfer method to safely treat patients with monogenic diseases.

Additional studies of gene therapy followed for other inborn errors of immunity (X-linked SCID, X-linked chronic granulomatous disease, and Wiskott-Aldrich syndrome), but were soon challenged with profound concerns when some of the patients involved in this treatment developed leukemia-like leukoproliferative conditions.^3^ The discovery of vector integrations near known proto-oncogenes in the leukemic clones halted the progress of viral vector based gene therapy.^4^^,^^5^ New lentivirus-based vectors were developed based on HIV-1; to minimize the risk of insertional oncogenesis, the strong enhancer elements of the viral long terminal repeats were eliminated in self-inactivating design. These lentiviral vectors exhibited a better safety profile and produced better clinical benefits due to their efficient gene transfer and stable expression in the modified cells demonstrated in long-term follow-up studies.^6^

The success of gene transfer is influenced by the viral vector’s ability to generate a functional copy of the DNA through reverse transcription. However, RT is highly error-prone without a proofreading function, as this contributes to the survival of the virus during infection by the generation of a swarm of quasi-species that can evade the immune response or anti-viral drugs.^7^^,^^8^ DNA copies with imperfect sequences are expected to integrate into the patient’s genome during gene therapy. The long-term consequences are often negligible when the RT generates synonymous mutations or missense mutations that result in a non-functional protein. However, certain genes can acquire gain-of-function and hyperactive mutations that can lead to malignant transformation.

Although other groups reported fidelity rates for HIV-1 and MLV RTs,^9^ a comprehensive analysis of mutational profiles of incorporated transgenes involved in past and current viral vector gene therapy trials has not been assessed. More recently, it has been suggested that apolipoprotein B mRNA-editing enzyme catalytic polypeptide-like (APOBEC) genes play key roles in mutagenesis during viral transduction.^10^

Duplex sequencing with targeted capture method was developed to detect low-frequency mutations.^11^ This approach is especially helpful for viral vector transduction protocols where a low copy number of the therapeutic transgene is the ideal outcome (e.g., 1–5 copies per cell). We investigated the vector transgene from transduced cells and distinguished variants generated by the action of RT and APOBEC3. In this study, we performed duplexing sequencing and a targeted capture method to investigate the *ADA* transgenes from the patients’ genomes, analyzing peripheral blood samples collected years after transplant with *ADA* gene vector modified CD34^+^ hematopoietic stem and progenitor cells.

Gene therapy for ADA-SCID has been one of the long-standing conditions studied in clinical trials of gene therapy to date, with one approach achieving approval by the European Medicines Agency in 2016.^1^^,^^12^ From trials of gene therapy for ADA-SCID performed at the University of California Los Angeles between 2009 and 2016, we obtained peripheral blood leukocyte samples from five patients treated with a γ-retroviral vector (MND-ADA), and five patients treated with a lentiviral vector (EFS-ADA).

Using this strategy, we (1) reported low-frequency mutations in the *ADA* transgenes from patients in two different clinical trials, (2) compared the fidelity between gamma-retrovirus and lentivirus RTs, and (3) identified a relatively higher frequency of mutations generated by the action of the APOBEC3 cytidine deaminases.

## Results

### Duplex sequencing of CD3^+^ cells transduced with ADA-SCID γ-retrovirus and lentivirus

**Figure 1:**
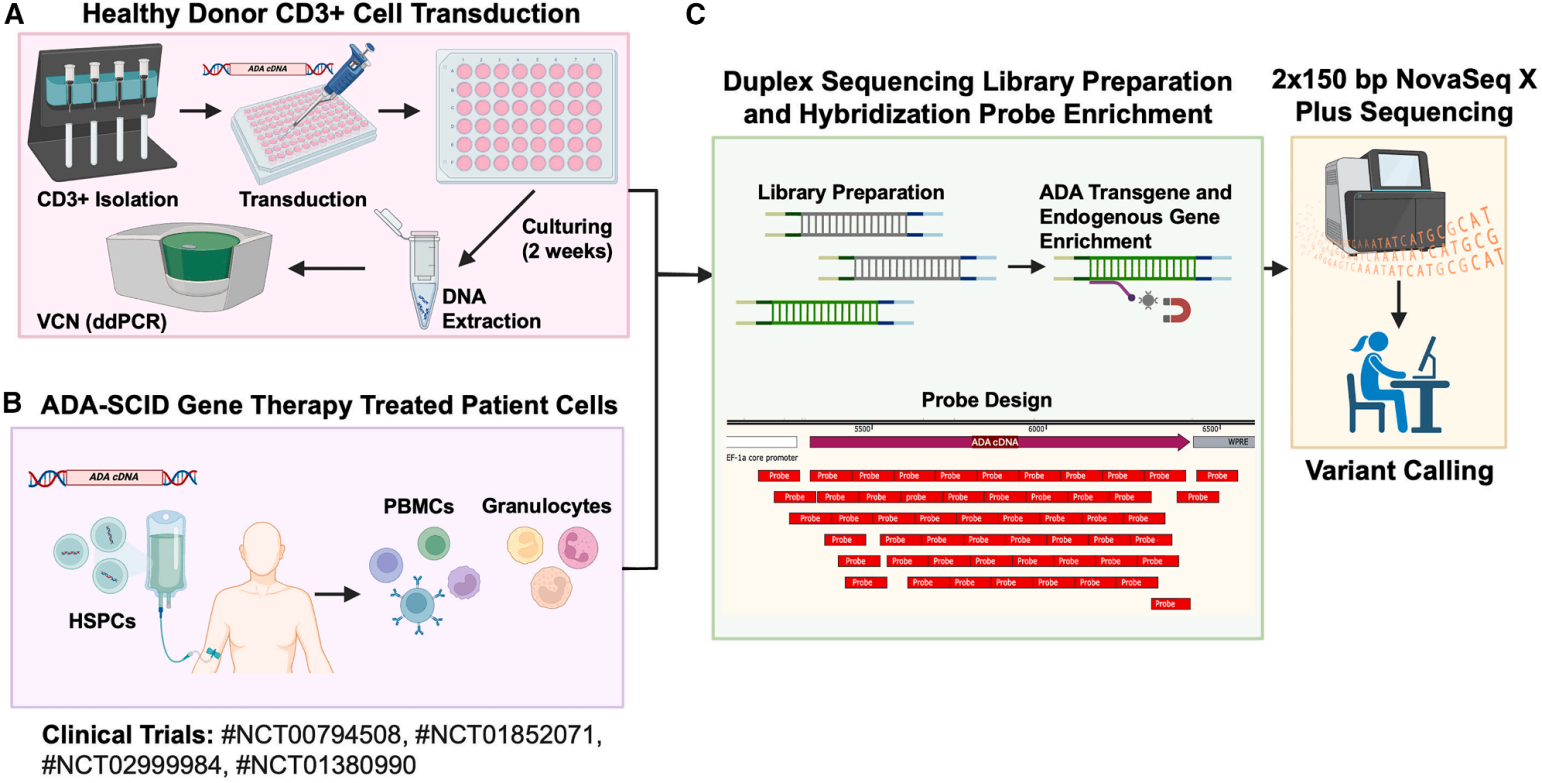
Duplex sequencing of healthy donor CD3^+^ cells and ADA-SCID gene therapy-treated patients; schematic of project protocol and duplex sequencing pipeline (A) CD3^+^ cells were isolated from healthy donor PBMCs and transduced at increasing viral volumes with either MND-ADA γ-retrovirus or EFS-ADA lentivirus. CD3^+^ cells were cultured for two weeks in T cell culture conditions. VCN of transduced cells were determined by ddPCR. (B) ADA-SCID gene therapy patient PBMCs and granulocytes were acquired from clinical trials #NCT00794508, #NCT01852071, #NCT02999984, and #NCT01380990. (C) Duplex sequencing library preparation protocol was performed on CD3^+^ and patient sample DNA. The *ADA* cDNA transgene and *ADA* endogenous gene were enriched through hybridization probe capture. Biotinylated probes were designed across the MND-ADA cDNA, EFS-ADA codon optimized cDNA, and across endogenous *ADA* exons. 2 × 150 paired end NovaSeq X Plus Sequencing was performed on the enriched DNA library. Variant calling was performed at an allele frequency of 0.1%. Image created with BioRender.

Before sequencing the vector-derived *ADA* transgene and the endogenous *ADA* gene from ADA-SCID patients’ peripheral blood mononuclear cells (PBMCs) and granulocytes, we optimized and assessed the sequencing pipeline to confirm the protocol was sensitive enough to detect the potentially rare mutations incorporated by RT. We acquired healthy donor PBMCs and isolated CD3+ T cells (Figure 1A). CD3^+^ cells were transduced with either the γ-retrovirus (MND-ADA)^2^ or the lentivirus (EFS-ADA)^13^ at increasing concentrations to obtain samples with a vector copy number (VCN) near to 1, to resemble the VCN of the patient PBMC samples. MND-ADA transduced CD3^+^ cells at a VCN of 0.7 and EFS-ADA transduced CD3^+^ cells at a VCN of 3.0 were selected for analyses.

A duplex sequencing library preparation protocol was selected due to the high sensitivity needed to detect rare variants and to distinguish true variants from errors incorporated through PCR amplification and sequencing, (Figure 1B). Duplex sequencing^11^ utilizes dual in-line unique molecular identifiers (UMIs) that are ligated to sheared DNA, which allows for the detection of errors incorporated during library preparation and sequencing. PCR amplification allows for the generation of two mirrored PCR products due to the dual UMIs. These products, post sequencing, can be error corrected by grouping these reads into two mirrored read families. If a mutation is seen in all the reads in both read families, this mutation was deemed true. Mutations that were only seen in a few reads of one read family were removed as false and likely to have been due to a mutation during PCR amplification or sequencing. Mutations seen in all the reads of one family but not the mirrored second read family were also called false and likely caused by a first-round PCR error.

An enrichment strategy was applied to the prepared library using biotinylated hybridization probes. These probes were designed to bind to the γ-retroviral and lentiviral *ADA* cDNA transgene as well as exons 2, 7, 8, 9, and 11 of the endogenous *ADA* gene. The enriched library was PCR amplified to produce the final library for sequencing.

CD3^+^ cell DNA was library prepped, enriched, and sequenced. FASTQ files were error corrected and false mutations were removed. For data processing and error correction, an error-correction protocol that minimizes false positives but requires high raw coverage was selected. Data were aligned to the human genome assembly GRCh38 (hg38) as well as to the MND-ADA and EFS-ADA vector sequences. Before and after error correction and read filtering, we analyzed sequencing statistics and alignments of the *ADA* transgene and endogenous *ADA*, as well as the coverage across the *ADA* cDNA (Table S1).

A single hybridization capture enrichment method versus a double hybridization capture enrichment method was first compared to assess the percentage of on-target reads and to see the effects on variant calling. A double hybridization capture method has been shown to have a higher percentage of on-target reads when paired with duplex sequencing library preparation.^14^ The data showed 30.44% and 41.58% on target alignments with a single hybridization capture and 43.26% and 98.70% on target alignments with a double hybridization capture in the MND-ADA and EFS-ADA samples before error correction (Table S1). While higher on-target reads were seen in the samples with double hybridization capture, the MND-ADA sample had a 7-fold decrease in data in comparison to the EFS-ADA sample with a more drastic difference in enrichment between the two samples (Table S1). During the hybridization capture step, the libraries are pooled together to optimize reagents, and we concluded that if samples have different VCNs of the target region (*ADA* transgene), a double capture leads to greater pull down of the sample with higher VCN leading to unequal sequencing and enrichment of the pooled samples. After error correction, collapsing reads, and filtering, most alignments (>90%) were aligned to the targeted regions of either the transgene or endogenous *ADA* regardless of using a single or double capture with all samples having high coverage (Table S1).

We then visualized the distribution of coverage across the transgene to make sure there was no drop-off of probes that could lead to low coverage in those regions. There was a uniform distribution across the EFS-ADA and MND-ADA transgene (Figures S1 and S2).

### Variant calling of transduced CD3^+^ cells

DNA variant calling was performed using an allele frequency of 0.1% for the transgene *ADA* and the endogenous *ADA* in the samples with single capture hybridization probe enrichment. Variant calling data showed a higher frequency of mutations in the lentiviral transduced sample (Table 1). There was also a high frequency of G-to-A mutations in the lentiviral sample, which was not seen in the γ-retroviral sample. The γ-retroviral sample had very few mutations consisting of only pyrimidine to pyrimidine base substitutions. Variants in the samples with double capture hybridization probe enrichment were also assessed and showed a decrease in called variants in the γ-retroviral sample and an increase in called variants in the lentiviral sample in comparison to the single capture samples (Table S2). While double capture may lead to greater sensitivity to detect rare sequence variants as shown in the lentiviral double capture sample, it can also potentially skew the variants due to unequal sequencing of samples, which can lead to losing rarer variants in the samples with lower VCN when pooled. Since the patient samples vary in VCN (Table S3) and will be pooled for the hybridization probe capture, we proceeded with a single capture to aim to prevent unequal sequencing and enrichment of samples with very low VCN.

**Table 1:** Transduced CD3^+^ cell transgene variants with single capture hybridization probe enrichment

	γ-Retrovirus (MND-ADA)	Lentivirus (EFS-ADA)
Total mutations	2	53
Purine to purine		
A to G	0	0
G to A	0	48
Pyrimidine to pyrimidine		
C to T	1	0
T to C	1	1
Purine to pyrimidine		
A to C	0	1
A to T	0	0
G to C	0	0
G to T	0	0
Pyrimidine to purine		
C to A	0	0
C to G	0	0
T to A	0	0
T to G	0	0
Deletion/insertion	0	1
Complex	0	2

The variants called in the endogenous *ADA* were then assessed to be used as a strategy to detect false mutations that were incorporated in the pipeline that were not caught by the error correction algorithm (i.e., DNA damage by thawing or sonication before UMI ligation). These variants in the exon and intronic regions detected were further analyzed in the SNP database (dbSNP)^15^ and all variants have been confirmed as either low-frequency somatic mutations or high-frequency SNPs seen in human populations (Table S4).

### ADA-SCID gene therapy-treated samples variant calling

Patient samples were obtained from clinical trials (#NCT00794508, #NCT01852071, #NCT02999984, and #NCT01380990) and duplex library prepped, sequenced, and variant calling was performed (Figure 1C). Patient sample sequencing statistics are shown in Tables S5 and S6. With this dataset, mutation profiles comparing the γ-retroviral-treated versus lentiviral-treated groups, as well as the mutational differences within the PBMCs and granulocytes were investigated.

Similarly to the CD3^+^ T cell data, there was a significantly higher frequency of mutations in the PBMCs lentiviral transduced samples (*p* = 1.2e−4) with a significantly greater frequency of G-to-A mutations in PMBCs (*p* = 9.9e−4) and granulocytes (*p* = 0.02). These mutations are seen at a low allele frequency (Table S7). The mutation profile of the γ-retroviral patient samples did not show high frequencies of specific types of mutations, but there were slightly higher rates of purine-to-purine and pyrimidine-to-pyrimidine mutations than any other type of single base substitutions. There were also significantly more complex mutations in the γ-retroviral treated patients’ granulocytes (*p* = 5.3e−3) in comparison with the lentiviral treated patients’ granulocytes (Table 2).

**Table 2:** Transgene DNA variants in γ-retroviral and lentiviral ADA-SCID gene therapy patients

Sample	γ-Retrovirus (MND-ADA)										Lentivirus (EFS-ADA)									
	Granulocytes					PBMCs					Granulocytes					PBMCs				
	402	404	405	408	410	402	404	405	408	410	501	504	509	511	512	501	504	509	511	512
Total mutations	11	6	14	9	7	8	8	10	14	21	77	15	68	61	20	75	75	106	76	97
Purine to purine																				
A to G	2	1	2	1	3	1	0	1	0	1	1	0	0	0	0	0	0	0	0	0
G to A	1	0	3	1	3	5	2	2	10	9	72	15	68	58	18	74	73	103	75	92
Pyrimidine to pyrimidine																				
C to T	1	1	3	1	2	1	2	1	2	2	1	0	0	2	1	0	2	0	1	2
T to C	2	1	0	1	2	0	0	2	0	2	0	0	0	0	0	0	0	1	0	1
Purine to pyrimidine																				
A to C	1	0	1	1	1	0	1	1	0	1	0	0	0	0	0	0	0	1	0	0
A to T	0	0	0	0	0	0	0	0	0	0	0	0	0	0	0	0	0	0	0	0
G to C	0	0	0	1	0	0	0	0	0	0	0	0	0	0	0	0	0	0	0	0
G to T	0	0	0	0	0	0	0	0	0	0	0	0	0	1	0	0	0	0	0	0
Pyrimidine to purine																				
C to A	0	0	0	0	0	0	0	0	0	0	0	0	0	0	0	0	0	0	0	0
C to G	1	1	0	1	1	0	1	1	0	1	0	0	0	0	0	0	0	0	0	0
T to A	0	0	0	0	0	0	0	0	0	0	1	0	0	0	0	0	0	0	0	0
T to G	0	0	0	0	0	0	0	0	0	0	0	0	0	0	0	0	0	0	0	0
Deletion/Insertion	0	0	0	0	1	0	0	0	2	3	0	0	0	0	1	0	0	0	0	2
Complex	3	2	5	2	4	1	2	2	0	2	2	0	0	0	0	1	0	1	0	0

**Figure 2:**
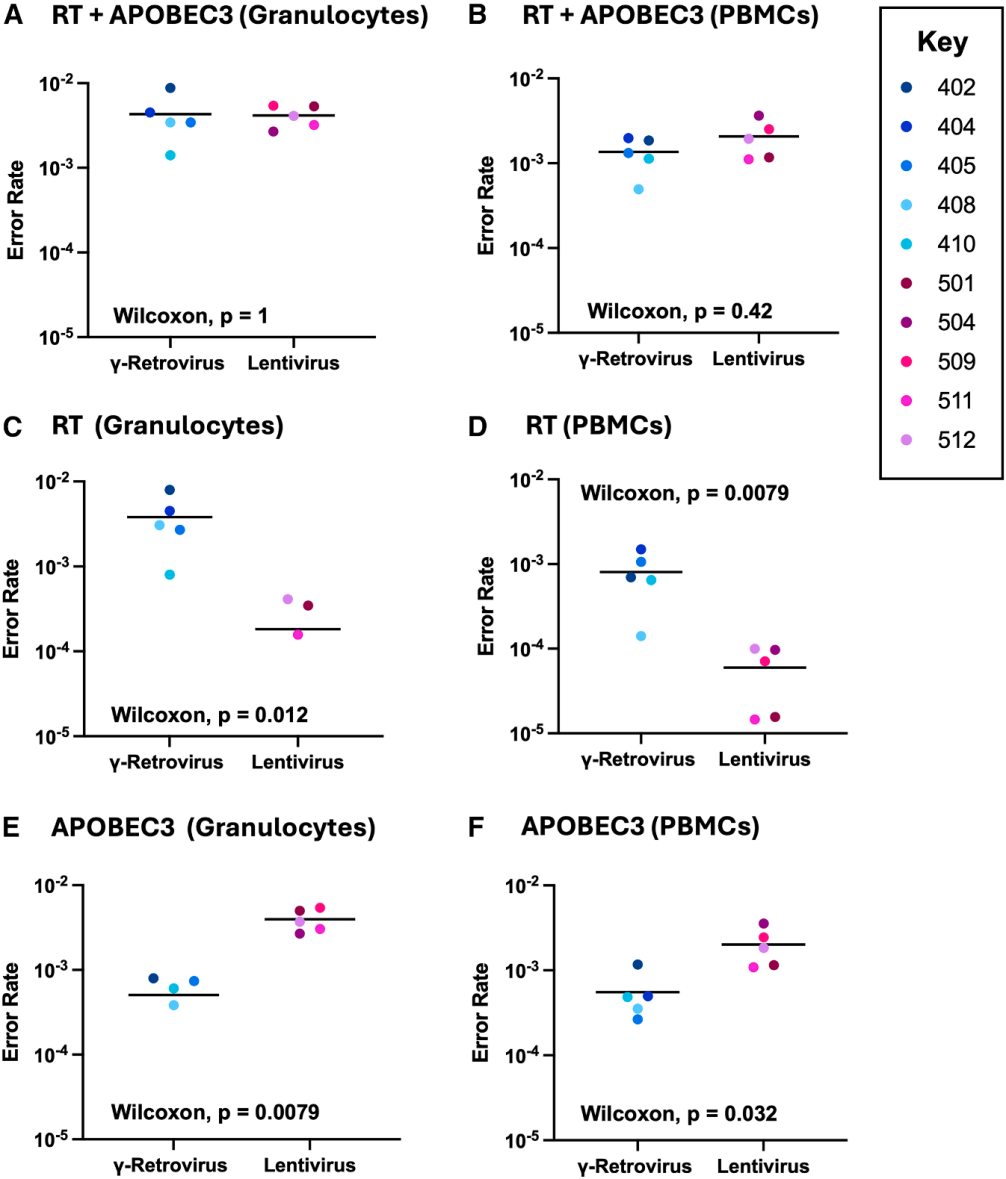
Error rates in *ADA* transgene in granulocytes and PBMCs in γ-retroviral and lentiviral ADA-SCID gene therapy patients Error rates were calculated as the number of mutations called divided by the number of reads aligned to the ADA transgene post error correction, read collapsing, and filtering, per patient sample. All G-to-A mutations were assumed to be caused by APOBEC3 and all other called mutations were deemed as RT mutations. (A) Error rate in granulocytes calculated with all mutations (RT + APOBEC3). (B) Error rate in PBMCs calculated with all mutations (RT + APOBEC3). (C) Error rate of RT in granulocytes. (D) Error rate of RT in PBMCs. (E) Error rate of APOBEC3 in granulocytes. (F) Error rate of APOBEC3 in PBMCs. Patient samples with no mutations in one of the categories (RT or APOBEC3) have no data point on the plot. A Wilcoxon rank-sum test was applied to compare the mean error rate between γ-retroviral and lentiviral treated samples (*n* = 5).

The error rates were calculated for RT and APOBEC3 in the γ-retroviral and lentiviral patient PBMCs and granulocytes. The assumption was made that all G-to-A mutations were due to APOBEC3 and all other mutations would be due to RT. The error rate was calculated as the number of mutations called divided by the number of reads aligned to the *ADA* transgene. Due to the patient samples having varying VCNs, calculating the mutation rate per read normalizes for *ADA* coverage across patient samples with different *ADA* transgene sequencing depths. We calculated an error rate for all mutations (RT + APOBEC3) as well as an error rate for RT only and for APOBEC3 only (Figure 2). While error rates for (RT + APOBEC3) were not significantly different between γ-retroviral and lentiviral patients’ granulocytes and PBMCs (Figures 2A and 2B), lentiviral RT had significantly lower mean error rates in both granulocytes and PBMCs (1.8e−4, 6.0e−5) in comparison with γ-retroviral RT (3.8e−3, 8.0e−4) (Figures 2C and 2D). APOBEC3 in the lentiviral patient samples had significantly higher mean error rates in both granulocytes and PBMCs (4.0e−3, 2.0e−3) in comparison with APOBEC3 in the γ-retroviral patient samples (5.0e−4, 5.5e−4) (Figures 2E and 2F).

The variants detected in the endogenous *ADA* gene in patient samples were then assessed (Table S8). Upon analysis, these were confirmed on the dbSNP and some of the variants that display a high frequency in the human population were also found across multiple patient samples (Table S9). Furthermore, the sequencing analysis was able to detect the reported pathogenic mutations for ADA-SCID (Figures S3–S10). In contrast, our analysis also detected variants and deletions that were not reported on the dbSNP database (Table S10). Most of these are intron variants, which may suggest recent somatic mutations generated throughout the patient’s lifetime or silent mutations from previous generations. Single nucleotide variants detected in the exons were confirmed to be synonymous protein mutations. However, our sequencing analysis detected large deletions that would be considered likely pathogenic as they result in a truncated ADA protein. Some of the large deletions were identified in at least two patient samples. Since our findings were not reported in any of the database, it is also possible that some of these are results of sequencing errors. Overall, our results support the efficacy of our sequencing pipeline to recognize and confirm different variants in the patient’s genome.

### ADA-SCID gene therapy-treated samples common DNA variants

**Figure 3:**
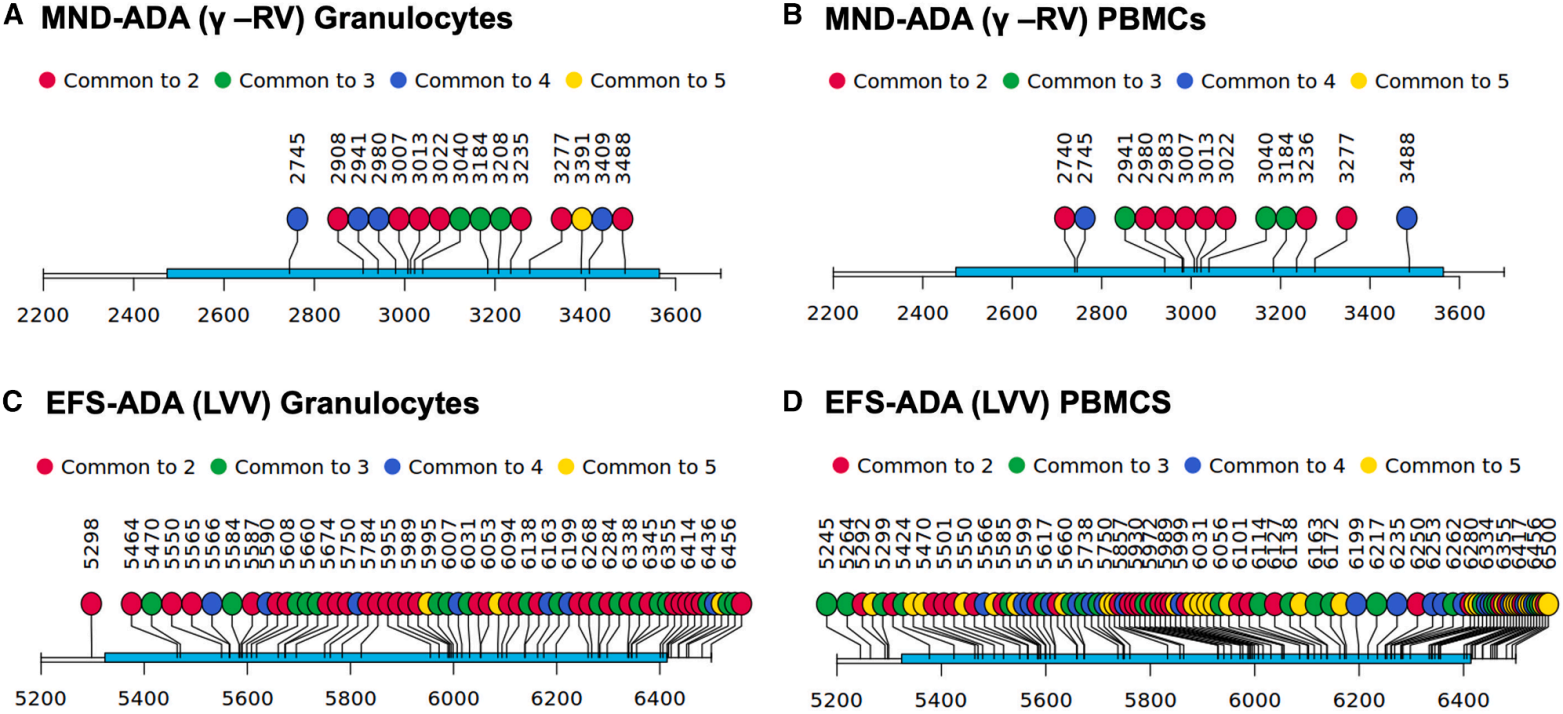
Common mutations in the *ADA* transgene were identified across patient samples Variants generated by gamma-retrovirus and lentivirus RT were called (allele frequency = 0.1%). Common mutations were observed among DNA samples from (A) granulocytes and (B) PBMCS from patients who received gamma-retrovirus gene therapy. (C) Granulocytes and (D) PBMCs from patients who received lentivirus gene therapy.

We next were interested to see if any of the variants called were common across the different patient samples. Four groups were created (five samples each consisting of the same viral treatment and cell type), and a high frequency of common variants were witnessed across the patient sample groups (Figure 3). Simulations were run to estimate the probability of observing repeated mutations in the transgene. We calculated the average number of mutations across five patients in each condition and generated random permutations of the mutation profiles using the mean observed mutation rates for 100,000 rounds, assuming each position had an equal probability of mutating. Then, the chances of seeing any given mutations at given number of times was computed. Any recurrent mutations with a probability of less than 0.05 are determined to be highly unlikely and may indicate a possible hotspot for mutations. Mutations common to three and four samples were shown to be highly significant (i.e., unlikely by chance) in the MND-ADA granulocytes (*p* = 0.002, *p* = 1e−05) and PBMCs (*p* = 0.005, *p* = 4e−05). Mutations common to four samples were shown to be significant in the EFS-ADA granulocytes (*p* = 0.009). While our simulations did not calculate the likelihood of observing mutations common to five samples, these events can be inferred as similarly unlikely in MND-ADA granulocytes and PMBCs, as well as EFS-ADA granulocytes.

### Validation of APOBEC3-mediated mutagenesis

To assess our hypothesis that the G-to-A signature could be due to the APOBEC3 protein family, a substrate analysis on the common variants in the lentiviral-treated patients was first performed to see if there was an indication of known APOBEC3 substrate sites.^16^ The substrate analysis showed a high frequency of mutations occurring at APOBEC3 substrate sites with a mean and standard deviation of 86.12 ± 4.89% (Table S11). We also predicted which APOBEC3 family protein may have been responsible for the deamination based on the substrate signature with A3D and A3H sites with high occurrence in PBMCs (Figure S11).

To further validate whether the observed G-to-A mutational signature was caused by APOBEC3-mediated mutagenesis, a HEK293T cell line was CRISPR edited to knock out APOBEC3 genes A through G. APOBEC3H was not targeted, as previous studies have reported no antiviral activity for this gene in HEK293T cells.^17^ While this is an incomplete knockout of all APOBEC3 genes, for these studies we will refer to this cell line as a “knock out.” The EFS-ADA vector was packaged in parallel using both parental HEK293T cells and APOBEC3 knock-out HEK293T cells. Concentrated viral preparations from each were used to transduce healthy donor CD34^+^ cells, which were then cultured under myeloid conditions for one week. Duplex sequencing of the *ADA* transgene was performed with sequencing information analyzed (Table S12) and variants were called using a 0.1% allele frequency threshold. Knocking out APOBEC3A-G genes in HEK293T cells resulted in a 91.2% reduction in G-to-A mutations along the EFS-ADA transgene in CD34^+^-transduced cells (Table 3).

**Table 3:** Transduced CD34^+^ cell transgene variants dependent on HEK239T packaging cell line

	Parental	APOBEC3 knockout
Total mutations	95	9
Purine to purine		
A to G	0	0
G to A	91	8
Pyrimidine to pyrimidine		
C to T	0	0
T to C	0	0
Purine to pyrimidine		
A to C	0	0
A to T	0	0
G to C	0	0
G to T	0	0
Pyrimidine to purine		
C to A	0	0
C to G	0	0
T to A	0	0
T to G	0	0
Deletion/insertion	1	1
Complex	3	0

### ADA-SCID gene therapy samples protein analysis

**Figure 4:**
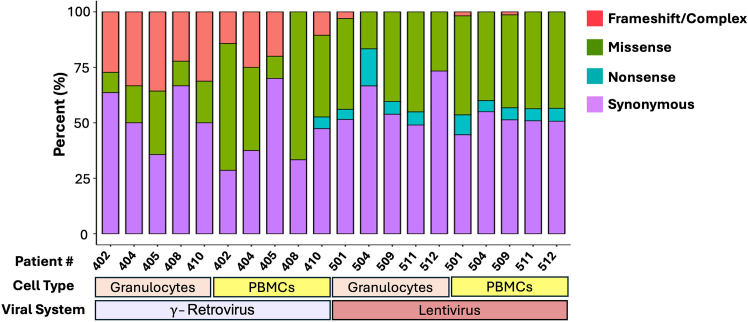
Characterization of *ADA* mutations based on encoded amino acid outcome Patient identification numbers in the 400s received γ-retrovirus gene therapy. Patient identification numbers in the 500s received lentivirus gene therapy. Only DNA mutations within the coordinates of *ADA* cDNA were analyzed. Percent was normalized by total mutations of each sample and called as either synonymous, nonsense, missense, or frameshift/complex.

We next analyzed how the DNA variants in each sample within the transgene would affect the coded ADA protein’s amino acid sequence (Figure 4). Within viral treatment type, there was a similar frequency of synonymous, missense, nonsense, and complex/insertion/deletion when comparing PBMCs to granulocytes (*p* values are nonsignificant). Comparing complex/insertion/deletion, we observed an increased number of mutations in granulocytes by γ-retrovirus (*p* = 2e−2). Comparing the lentiviral treated samples to the γ-retroviral treated samples, there was a decrease in missense in granulocytes (*p* = 3.1e−2) and an increase in complex/insertion/deletion (*p* = 1.4e−4) in the γ-retroviral treated samples in comparison with the lentiviral samples. In PBMCs, we observed an increased mutation rate in nonsense (*p* = 5.3e−3) and a decrease in frameshift (*p* = 3.5e−2) in lentiviral-treated samples.

### Evaluation of mutational landscape over time

The mutational landscape of the EFS-ADA transgene was assessed in samples from two additional ADA-SCID lentiviral gene therapy patients, to determine if potentially deleterious mutations would be selectively depleted over time. The DNA from the drug product (EFS-ADA transduced CD34^+^ cells) and PBMCs from two time points post gene therapy (Table S13). T cell DNA from each of these patients was also assessed due to the potential of T cells having greater selective pressure due to high ADA expression. The samples were duplex sequenced and variant calling was performed at an allele frequency threshold of 0.01%. Due to higher sequencing coverage across the EFS-ADA transgene in these patient samples, we had more confidence to call variants at a lower allele frequency than the 0.1% allele frequency used for previous datasets shown in this study (Table S14).

The data revealed consistently high frequencies of G-to-A mutations across all samples (Table S15). To evaluate whether deleterious mutations were selectively depleted over time due to reduced cellular fitness, variant allele frequencies were compared longitudinally (Figures S12–S15). An amino acid level analysis was conducted to assess the percent distributions of synonymous, missense, and complex mutations (Table S16). However, the relative frequencies of mutation types remained similar across all time points and cell types, suggesting these mutations are present in the drug product and then stable.

### Assessment of APOBEC3-mediated mutation occurrence

**Figure 5:**
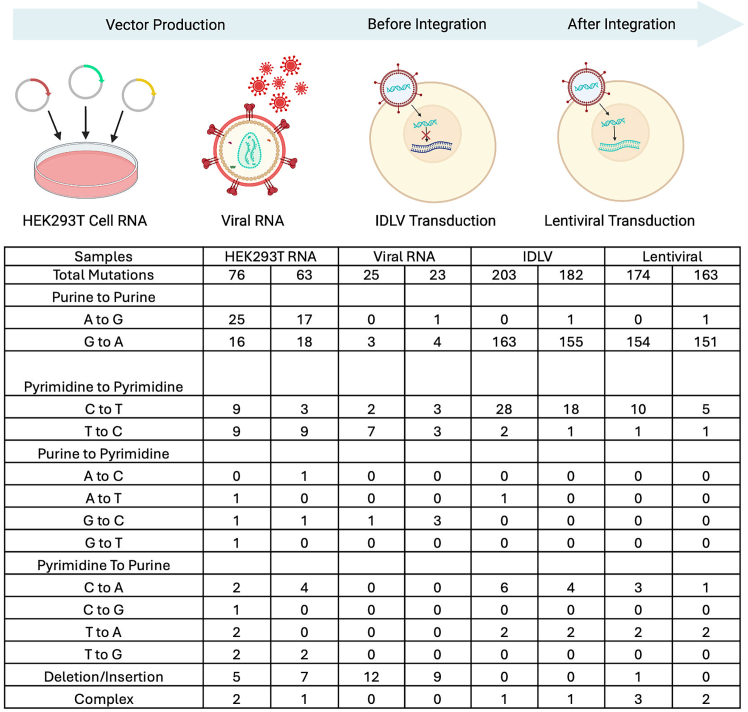
Mutational landscape of APOBEC3 mutation occurrence from EFS-ADA vector packaging to transgene integration HEK293tRNA from cells during packaging, viral RNA, and DNA from CD34^+^ transduced with IDLV and lentivirus were library prepped and sequenced. Variant calling was performed at an allele frequency of 0.01%. Image created with BioRender.

We conducted a duplex sequencing experiment to determine the timing of APOBEC3-related mutations. In duplicate, we sequenced RNA from parental HEK293T packaging cells three days post-transfection with the EFS-ADA LV plasmid, as well as RNA from purified virion preparations, to evaluate mutations arising during vector production. To assess mutations prior to integration, EFA-ADA integrase-defective lentiviral vectors (IDLVs) were packaged, and DNA was collected from CD34^+^ transduced cells 48 h post-transduction. To evaluate post-integration mutations, CD34^+^ cells were also transduced with EFS-ADA lentivirus and cultured for two weeks. Due to high sequencing coverage (Table S17), variants were called at an allele frequency threshold of 0.01% across all samples. G-to-A mutations were observed in all conditions, with the highest frequencies detected in CD34^+^ cell target samples transduced with EFS-ADA IDLV and lentivirus (Figure 5). Although some APOBEC3-related mutations may occur during vector production, the data indicates that the majority arise before integration.

## Discussion

While fidelity and mutational profiles of RTs (HIV-1 and M-MLV) have been previously studied, there has been little research on RT fidelity in retroviral and lentiviral gene therapy. Gene therapies that utilize these vectors may be introducing unwanted genetic changes to the incorporated transgene due to errors from RT. While there are some genes where the incorporation of a disruptive mutation will leave the protein non-functional like ADA, there are many other diseases and malignancies where the incorporation of the mutation in a transgene can lead to oncogenesis. For example, an essential cytokine receptor for T cell development, IL7Rα can cause SCID when defective and is thus an important target for gene therapy but, activating mutations of *IL7Rα* may be driver mutations in T cell leukemias.^18^ With this being the case, it is important to understand mutational profiles to make sure that retroviral and lentiviral approaches are safe for gene therapy for certain diseases and malignancies.

With unique access to ADA-SCID gene therapy patients’ PMBCs and granulocytes, we performed duplex sequencing of the *ADA* cDNA transgene as well as the endogenous *ADA* gene to better understand the mutational landscapes in gene therapy using retroviral and lentiviral delivery. Deep sequencing was performed, which produced high raw coverage in the targeted regions, which allowed for the utilization of a stringent error correction bioinformatic protocol to remove any false mutations incorporated into the pipeline. After sequencing, data processing, and variant calling, the data illuminated a significantly higher mutation signature of G-to-A mutations in the lentiviral vector treated T cells and patient samples. We hypothesized that this mutational signature may not be due to RT but to the APOBEC3 protein family. APOBEC3 is a cytidine deaminase that is part of the innate immune response that causes mutations in viral RNA or ssDNA during reverse transcription to prevent viral replication and infectivity.^19^^,^^20^ By deaminating cytosines, which are then converted to uracil or thymine, APOBEC3 will lead to DNA guanine to adenosine transitional mutations.

The fidelity of RT in both γ-retroviral and lentiviral-treated patient cells was next calculated. While it has been shown that MLV RT typically has a higher fidelity than HIV-1 RT,^21^ our patient dataset showed that HIV-1 RT exhibited a greater fidelity. Some explanations for this could be the assumption made for the calculation that all G-to-A mutations were due to APOBEC3 when some may have been caused by RT. There also may have been mutations missed due to selective advantage in our PBMC population. For example, if a cell received a single *ADA* transgene (VCN = 1) with a mutation deeming it non-functional, that cell would not survive and therefore those mutations would be missed in our analysis, leading to greater fidelity.

We also investigated if similar mutations were present across the different patient samples or if mutations occurred at random. While the γ-retroviral samples had far fewer mutations than the lentiviral samples, recurrent mutations across samples were observed at multiple sites, which is unlikely to be due just to chance but may represent hot spots for RT errors or APOBEC activity. A high frequency of common recurrent mutations in the lentiviral samples was also witnessed.

We next analyzed the effects of the mutated allele on the coded amino acid of the ADA protein were analyzed. We had hypothesized that we may see a higher proportion of synonymous protein mutations in the PMBCs than in the granulocytes in both the lentiviral and y-retroviral samples because the lymphocytes (T, B, and natural killer cells) among the PBMC fraction need a functional copy of *ADA* to survive, while granulocytes do not, based on the observed strong selective advantage for ADA gene replete lymphocytes after gene therapy, but not for granulocytes.^2^

To our surprise, we did not see a higher frequency of synonymous mutations in the PBMCs when compared with granulocytes within viral treated groups. One explanation for this is that the cells containing a missense, nonsense, or frameshift/complex mutation in the incorporated transgene, may also have an additional functional copy of the *ADA* transgene with no mutations incorporated. Their only needs to be one functional copy of the *ADA* gene within the lymphoid cells for normal immune function as seen in heterozygous carriers who are asymptomatic.

A DNA substrate analysis of the G-to-A mutations in the lentiviral samples was performed to assess if there were any known APOBEC3 substrate sites at these mutation locations. APOBEC3 substrate sequences^16^ were utilized and confirmed a high frequency of 86.12 ± 4.89% across all lentiviral samples, showing that the majority of G-to-A mutations contained an APOBEC3 substrate sequence. We further validated the hypothesis that the G-to-A transitions seen in the transgene are due to the activity of APOBEC3 by knocking out APOBEC3 genes in HEK293T cells. The data showed 91.2% reduction in G-to-A mutations in CD34^+^ cells transduced with lentiviral preparations packaged in the APOBEC3 knockout cell line, indicating that this mutational signature is largely attributable to APOBEC3 activity derived from the packaging cell line.

The endogenous *ADA* gene was sequenced to define the baseline error rate in our pipeline for false mutations that were not corrected with the error correction protocol. We should only see the known pathogenic *ADA* mutations in the exons of each patients’ samples that were their cause for ADA-SCID; all other exonic mutations could be used to create a baseline error rate. What we came to realize is that due to the hybridization capture, we sequenced exon and parts of the intronic regions leading to the detection of low frequency alleles. We investigated these alternate alleles (majority in intronic regions) and saw that most of these alleles have been cited and seen and documented in global populations and therefore concluded that these could be true alleles carried by these individuals or low-frequency somatic mutations. In addition, common variants that were identified in multiple patient samples further suggest that these are more true variant alleles than sequencing errors. We anticipate the new variants and large deletions that were not reported on any database can either be low frequency somatic mutations or sequencing errors. Through the detected current and potentially new variants that were identified in at least two patient samples, this established sequencing pipeline can distinguish changes in both the patient’s genome and transgene.

We also questioned why we were not seeing APOBEC3-mediated restriction in our γ-retroviral samples. Both viruses have evolved methods to counteract APOBEC3 to better preserve their genome integrity. MLV has two mechanisms to inhibit APOBEC3 activity that could be the reasons as to why we see minimal G-to-A mutations in our data. MLV produces an alternatively spliced P50 protein from the *gag* RNA, which prevents APOBEC3 from being packaged into the viral particles during packaging.^22^ MLV also produces a glycosylated Gag (glycoGag) protein which prevents APOBEC3 interaction with the reverse transcription complex due to increasing stability of the viral core.^22^ HIV-1 expresses the accessory protein VIF that counteracts the activity of APOBEC3, but standard lentiviral vector packaging systems have excluded the accessory proteins including VIF as they were not thought to be essential for vector production and it is important to minimize HIV-1 components in the vector production systems for biosafety.

Evolution of the mutational landscape over time was then assessed in two additional lentiviral vector gene therapy patients. While relative frequencies of mutation types remained similar across all time points, the stability suggested that if deleterious mutations are present, they may not significantly impact cell survival, or potentially affected cells also carry additional copies of the functional ADA transgene (VCN ≥ 2). In such cases, the deleterious effect may be compensated, allowing the mutations to persist over time. Given the consistent mutation frequencies, there is no clear evidence of global selective pressure against these variants. We also concluded that variants observed exclusively in the drug product or only in the post-gene therapy DNA samples could be because the sequenced drug product was a retention sample, rather than the actual material administered to the patient. As a result, variants present in the transplanted material may not have been captured in the sequenced retention sample, and vice versa.

Finally, we sequenced RNA from packaging cells and viral particles along with DNA from CD34^+^ cells transduced with IDLV and lentivirus to assess when these APOBEC3-mediated mutations occur. The data showed a jump in G-to-A frequency in the IDLV transduced samples indicating that APOBEC3 activity is primarily occurring between viral entry into the target cell and the end of the reverse transcription process. While we did see some G-to-A mutations in HEK293T RNA, these mutations most likely are due to the virus being produced and released into the medium, reinfecting the cells, and mutagenized by APOBEC3 in the HEK293T cells, producing the altered *ADA* mRNA. Alternatively, the activity of APOBEC3 protein prefers RNA mutagenesis that could then be detected by RNA sequencing.

Because APOBEC3-derived mutations were the predominant error we observed in lentiviral vector proviruses, efforts to reduce APOBEC3 activity could produce more sequence consistency for clinical lentiviral vectors. Approaches to be explored include adding a VIF-expression plasmid during packaging or deleting the APOBEC3 gene complex from the packaging cells for the development of virion lacking APOBEC3 proteins.

## Materials and methods

### Vector production

The EFS-ADA and MND-ADA vector cloning design have been described previously.^13^ HEK293T *PKR* knockout cells^23^ were plated on 10-cm plates at a density of 1 × 10^7^ cells/mL and vectors were packaged through transient transfection. EFS-ADA was packaged using third generation lentiviral packaging plasmids using the VSV-G protein envelope. Raw viral supernatant was collected 3 days post transfection and concentrated through ultracentrifugation. MND-ADA was packaged using MLV-based packaging plasmids^24^ and VSV-G protein. Raw viral supernatant was collected 3 days post transfection and concentrated using Retro-X Concentrator (Takara Bio Inc., Shiga, Japan).

### Healthy donor T cell (CD3^+^) transduction and culturing

Healthy donor PBMCs were acquired from the UCLA Virology Core. T cells (CD3^+^) were selected from PBMCs using the CD3 Microbead kit (Miltenyi Biotec, Auburn, CA, USA). CD3^+^ cells were plated at 1.5 × 10^6^ cells/mL and stimulated with Immunocult CD3/CD28 activating cocktail (STEMCELL Technologies, Vancouver, Canada). We plated 72 h post activation cells on retronectin-coated plates at a concentration of 1 × 10^6^ cells/mL in X-Vivo 15 medium (Lonza, Basel, Switzerland) supplemented with 1× glutamine, penicillin, and streptomycin (Gemini Bio-Products, Sacramento, CA, USA), 1× Glutamax (Thermo Fisher Scientific, Irwindale, CA, USA), 5% human AB serum (Gemini Bio-Products, Sacramento, CA, USA), 100 U/mL human interleukin-2 (Peprotech, Rocky Hill, NJ, USA), and transduced with ascending amounts of vector (1, 3, 5, 10, 30, and 50 μL) and transduction enhancers Poloxamer 338 (1 mg/mL) (BASF, Ludwigshafen, Germany) and Prostaglandin E2 (10 μM) (Millipore Sigma, Burlington, MA, USA). Cells were cultured for two weeks and collected on day 14.

### VCN analysis

VCN was determined from extracted genomic DNA for samples transduced with EFS-ADA by digital droplet PCR (ddPCR) assay using primers and probes for HIV-1 PSI (forward 5′-AAGTAGTGTGTGCCCGTCTG-3′, reverse 5′-CCTCTGGTTTCCCTTTCGCT-3′, 5′-56-FAM-CCCTCAGAC-ZEN-CCTTTTAGTCAGTGTGGAAAATCTCTAG-3IABkFQ-3′). VCN was determined for samples transduced with MND-ADA by ddPCR assay using primers and probes for human ADA cDNA (forward 5′-GGTCCATCCTGTGCTGCAT-3′, reverse 5′-CGGTCTGCTGCTGGTACTTCTT-3′, 5′-56-FAM-CCAGCCCAA-ZEN-CTGGTCCCCCAAG-3IABkFQ-3′). The Human Syndecan 4 gene (SCD4) as a reference (forward 5′-CAGGGTCTGGGAGCCAAGT-3′, reverse 5′-GCACAGTGCTGGACATTGACA-3′, 5′-5HEX-CCCACCGAA-ZEN-CCCAAGAAACTAGAGGAGAAT-3IABkFQ-3′) for all samples.

### RNA extraction and cDNA synthesis

RNA was extracted from HEK293T cells three days post transfection of the packaging plasmids using the RNeasy Mini Kit (Qiagen, Hilden, Germany). Viral RNA was extracted from unconcentrated EFS-ADA viral supernatant using the QIAamp Viral RNA Kit (Qiagen). All RNA samples were DNASE I (Invitrogen, Waltham, MA, USA) digested for 15 min, and then heat inactivated. First strand synthesis was performed using the SuperScript III First-Strand Synthesis Kit (Invitrogen). The second strand was synthesized by 1× cycle PCR using Platinum SuperFi II Green PCR Master Mix (Invitrogen). PCR product was purified for library preparation using AMPureXP beads (Beckman Coulter, Brea, CA, USA).

### Library preparation, target hybridization, and DNA sequencing

DNA and cDNA libraries were prepared using the xGen cfDNA & FFPE DNA Library Prep v2 MC Kit (Integrated DNA Technologies, San Diego, CA, USA). For each sample, 250 ng of genomic DNA were processed. All DNA samples were sheared using the M220 Focused-ultrasonicated (Covaris, Woburn, MA, USA) to create fragments with an average size of 250 base pairs. The end repair, adapter ligation and PCR amplification were performed according to the manufacturer’s manual. The adapters contain 8-bp UMIs with optimized and fixed sequences (32). Because fixed sequences were used, any errors generated from sequencing or PCR can still be corrected during the analysis. Barcodes were introduced during PCR amplification (six cycles) using xGen unique Dual Index primer pairs (Integrated DNA Technologies). For the enrichment of the library, a custom panel of probes was designed to target both codon-optimized and wild-type sequence of the ADA gene. Hybridization capture was performed with xGen Hybridization and Wash v2 Kit (Integrated DNA Technologies), xGen Universal Blockers TS (Integrated DNA Technologies), and xGen Library Amplification Primer Mix (Integrated DNA Technologies), with an input of 1 μg of DNA library for each capture. Duplex sequencing was performed with the Illumina NovaSeq X Plus (2 × 150).

### Variant calling

The Integrated DNA technologies xGen cfDNA & FFPE DNA Library Prep Kit “Processing sequence data with UMIs analysis guidelines” (xGen cfDNA & FFPE DNA Library Prep v2 MC Kit, #10010206, IDT, San Diego, CA, USA) was used for data processing and variant calling. Unmapped BAM files were constructed from demultiplexed FASTQ files using Picard^25^ version 3.0 UMIs were extracted using fgbio version 2.2.1 with a “--read-structure = 8M142T 8M142T.” Reads were aligned using bwa^26^ version 0.7.18 (r1243) to the reference genome hg38 and either MND-ADA or EFS-ADA reference sequences. We selected the collapsing combined read families error correction method. VarDictJava^27^ version 1.8.3 was used to call variants with an allele frequency of 0.1%. Allele frequency of 0.01% was used to call variants for mutation landscape over time and APOBEC3 mutation occurrence experimentation. All other commands and parameters were based on suggested parameters from the analysis guidelines.

### APOBEC3 substrate analysis

APOBEC3 substrates were obtained from Maiti et al.^16^ and G-to-A mutations were analyzed by comparing DNA sequence at the G-to-A site with APOBEC3 substrate sequences.

### Statistics

A *t* test was applied to the patient variant callset to compare PMBCs and granulocytes, as well as γ-retroviral and lentiviral treated samples. To assess the likelihood of observing variants common to multiple samples, we ran simulations to estimate the probability of observing repeated mutations in the transgene. We calculated the average number of mutations across five patients in each condition and randomly generated mutations using the empirical mutation rates for 100,000 rounds, assuming each position had an equal probability of mutating. Then, we computed the chances of seeing any given mutations a given number of times. Any sites with a given number of mutations are determined to be highly unlikely if the simulated rates are less than 0.05. A Wilcoxon test was used in comparing the means of error rates between groups. This non-parametric test does not assume an underlying statistical distribution and a *p* value of less than 0.05 shows that the error rate is significantly higher in one group than the other.

## Data availability

Data available from Dr. Donald B. Kohn upon request. DNA sequencing data are available at NCBI: PRJNA1252794 (National Center for Biotechnology Information Sequence Read Archive under BioProject accession number PRJNA1252794).

## Acknowledgments

These studies were supported by endowment funding from the UCLA Eli & Edythe Broad 10.13039/100015705Center of Regenerative Medicine and Stem Cell Research (D.B.K.). Training grants provided support to K.L.H. (10.13039/100000002NIH TL1 DK132768 and U2C DK129496). The UCLA Clinical Translational 10.13039/501100020584Science Institute provided funding for sequencing through the T1/T2 Accelerator Program Core Voucher Award (10.13039/100000002NIH/National Center for Advancing Translational Science UCLA CTSI Grant UL1TR001881). The UCLA/CFAR Virology Core Lab (5P30 AI028697) provided healthy donor PBMCs.

## Author contributions

D.B.K. conceived of this study. K.L.H., R.C., and K.B. designed experiments. K.L.H., R.C., and A.H.M. executed experiments. N.K. helped execute portions of experiments. K.L.H. analyzed experiments. K.L.H. and L.Z. performed bioinformatic analyses and statistics. D.B.K. advised experiments. K.L.H., R.C., and D.B.K. wrote the manuscript.

## Declaration of interests

D.B.K. is an inventor for the UC Regents of the EFS-ADA lentiviral vector.

## Supplemental information

Document S1. Figures S1–S15 and Tables S1–S6 and S8–S17

Table S7. Allele Frequencies of G to A Mutations in Lentiviral Treated Patient Cells

Document S2. Article plus supplemental information
