# Supplementary material for: Quantifying the mutational landscape of retroviral and lentiviral vectors in gene therapy patients
Source: Mol Ther. 2025 May 30;33(9):4459–70. doi: 10.1016/j.ymthe.2025.05.034 (PMC12432904; doi:10.1016/j.ymthe.2025.05.034)
Supplement: Supplementary file 1 [file mmc1.pdf]

YMTHE, Volume *33*

Supplemental Information

Quantifying the mutational landscape of retroviral and lentiviral vectors in gene therapy patients

Kevyn L. Hart, Ralph Valentine Crisostomo, Annika Mittelhauser, Lingyu Zhan, Nika Kononov, Kathryn Bradford, and Donald B. Kohn

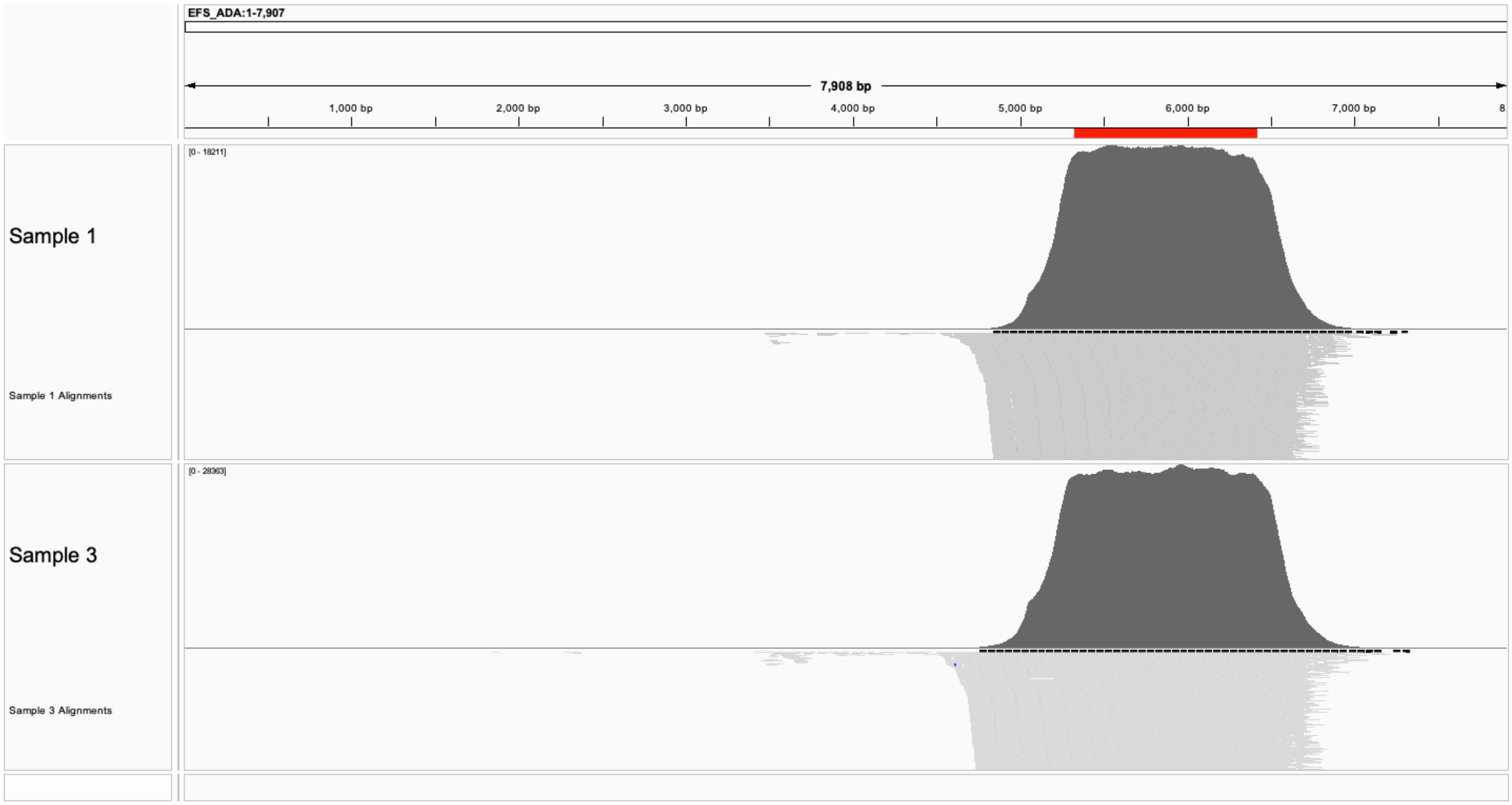

Figure S1: Alignment and Coverage Distribution of EFS-ADA cDNA
Integrative Genomics Viewer image of sample 1 and 3 alignments and coverage distribution of the EFS-ADA transgene. The red bar indicates the location of the ADA cDNA showing high coverage and uniform distribution across the cDNA.

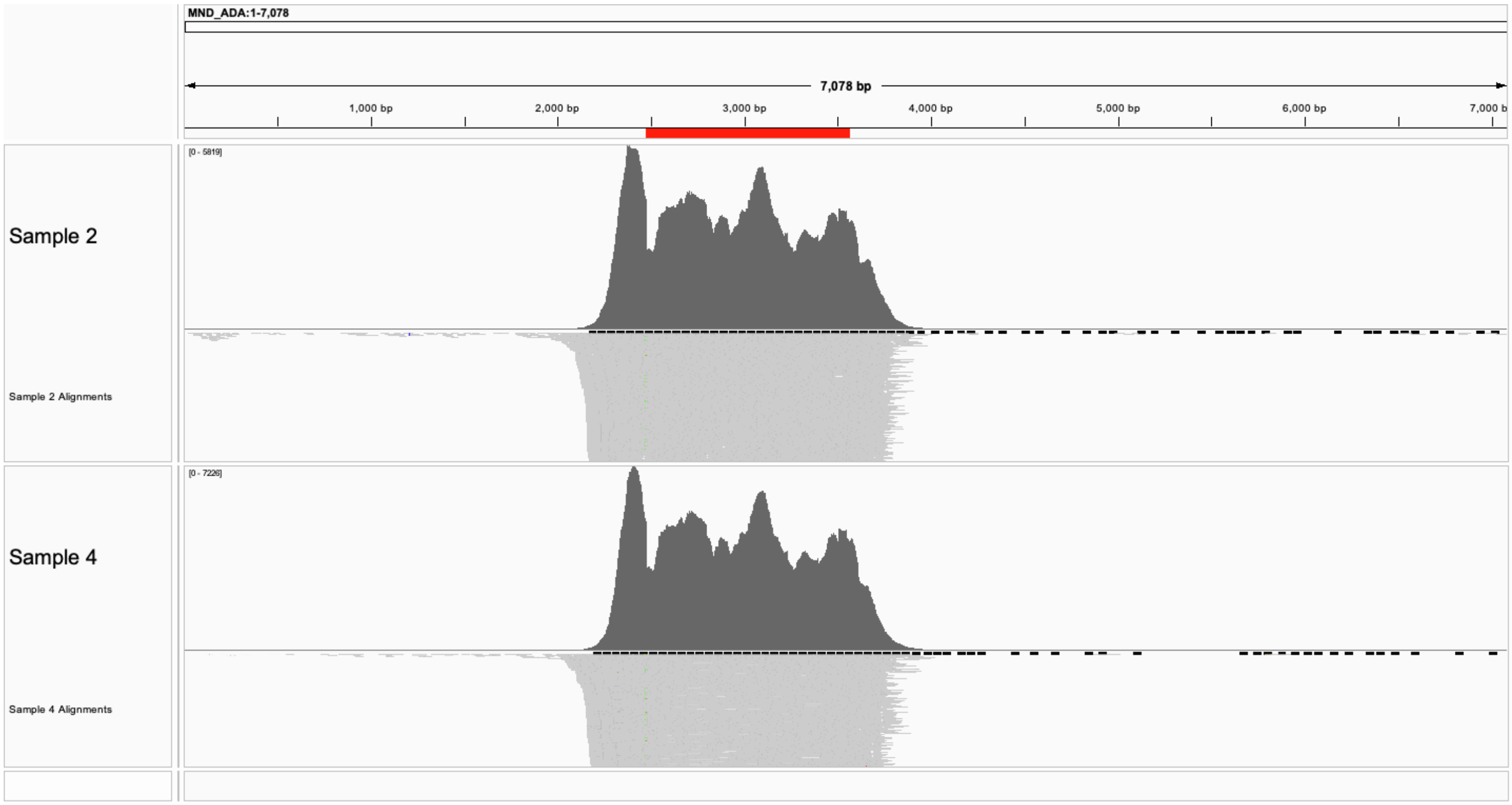

Figure S2: Alignment and Coverage Distribution of the MND-ADA retroviral vector *ADA* cDNA Integrative Genomics Viewer image of sample 2 and 4 alignments and coverage distribution of the MND-ADA transgene. The red bar indicates the location of the ADA cDNA showing high coverage across the cDNA.

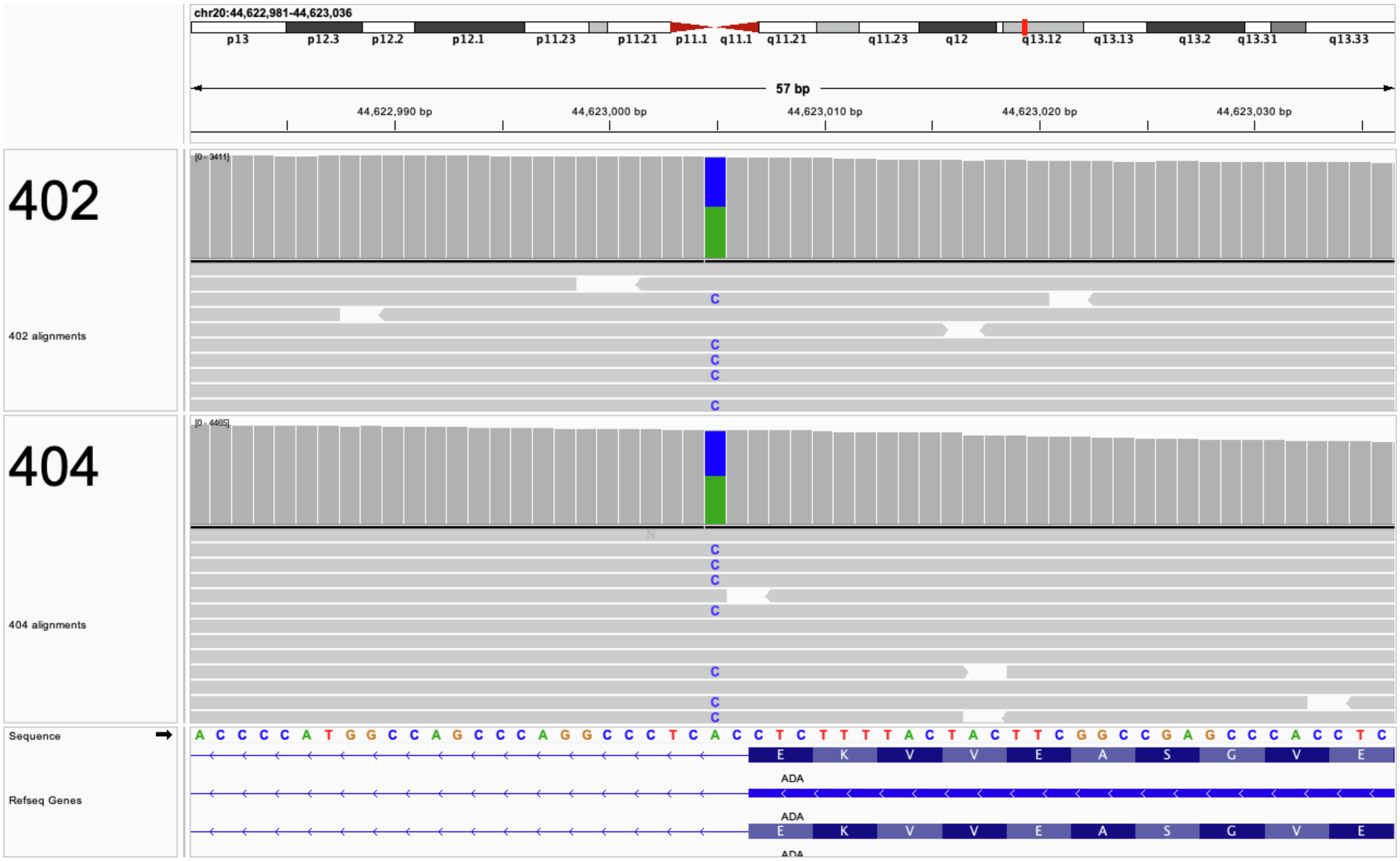

Figure S3: Visualization of Patients 402 and 404 ADA-SCID Mutations (T+2>G in IVS7)
Integrative genomics viewer visualization of ADA-SCID mutations.

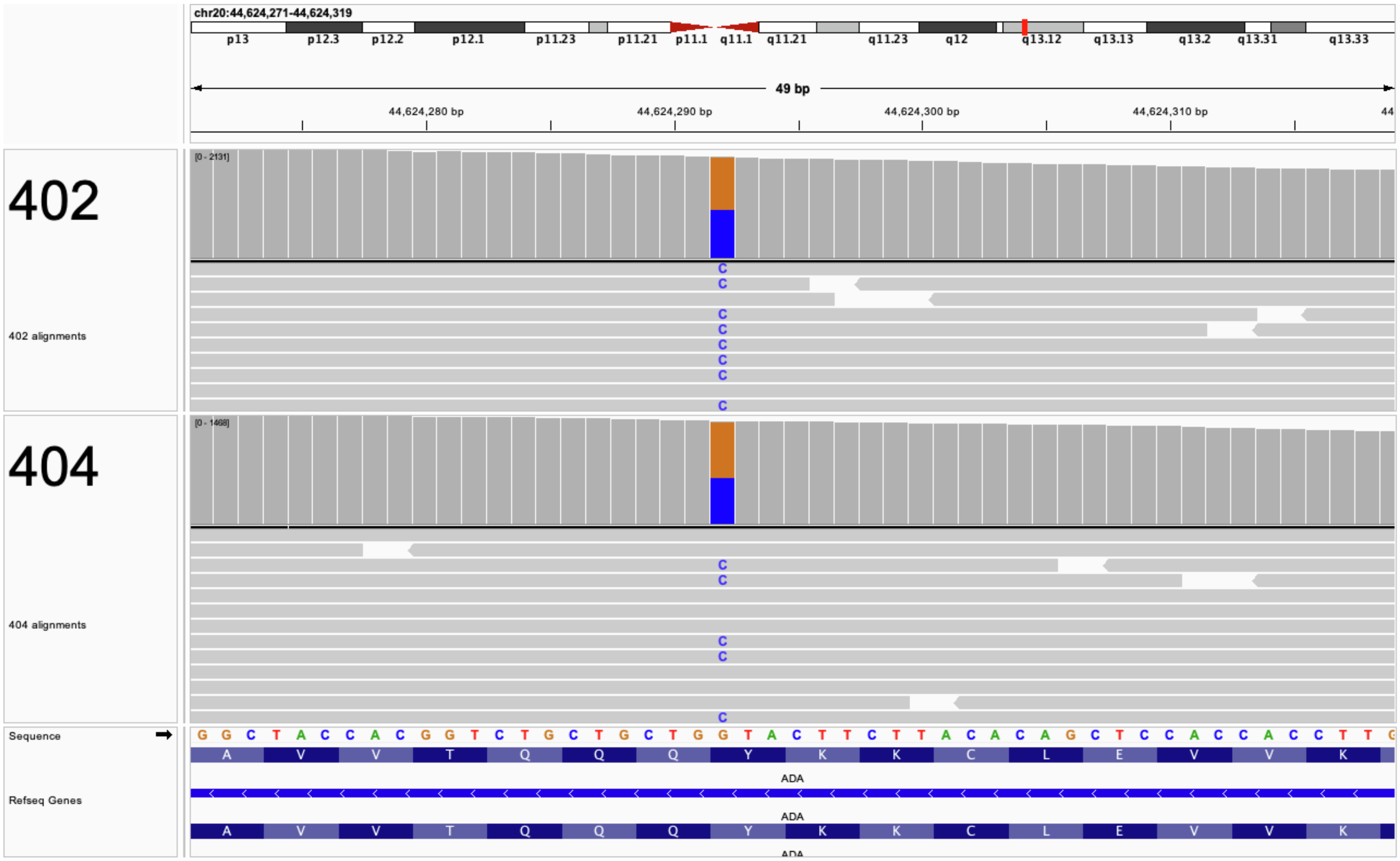

Figure S4: Visualization of Patients 402 and 404 ADA-SCID Mutations (Y172STOP)
Integrative genomics viewer visualization of ADA-SCID mutations.

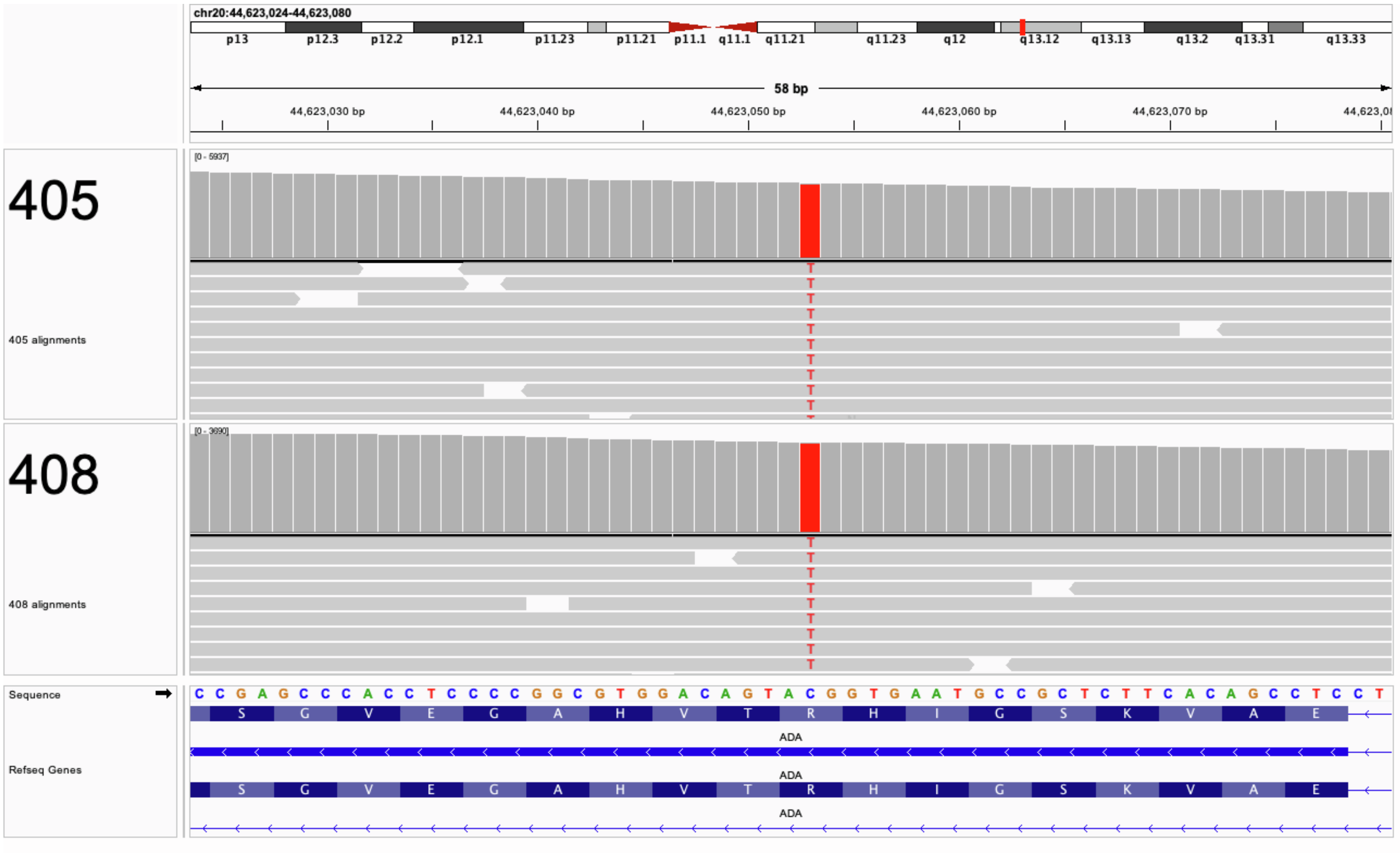

Figure S5: Visualization of Patients 405 and 408 ADA-SCID Mutations (R211H)
Integrative genomics viewer visualization of ADA-SCID mutations.

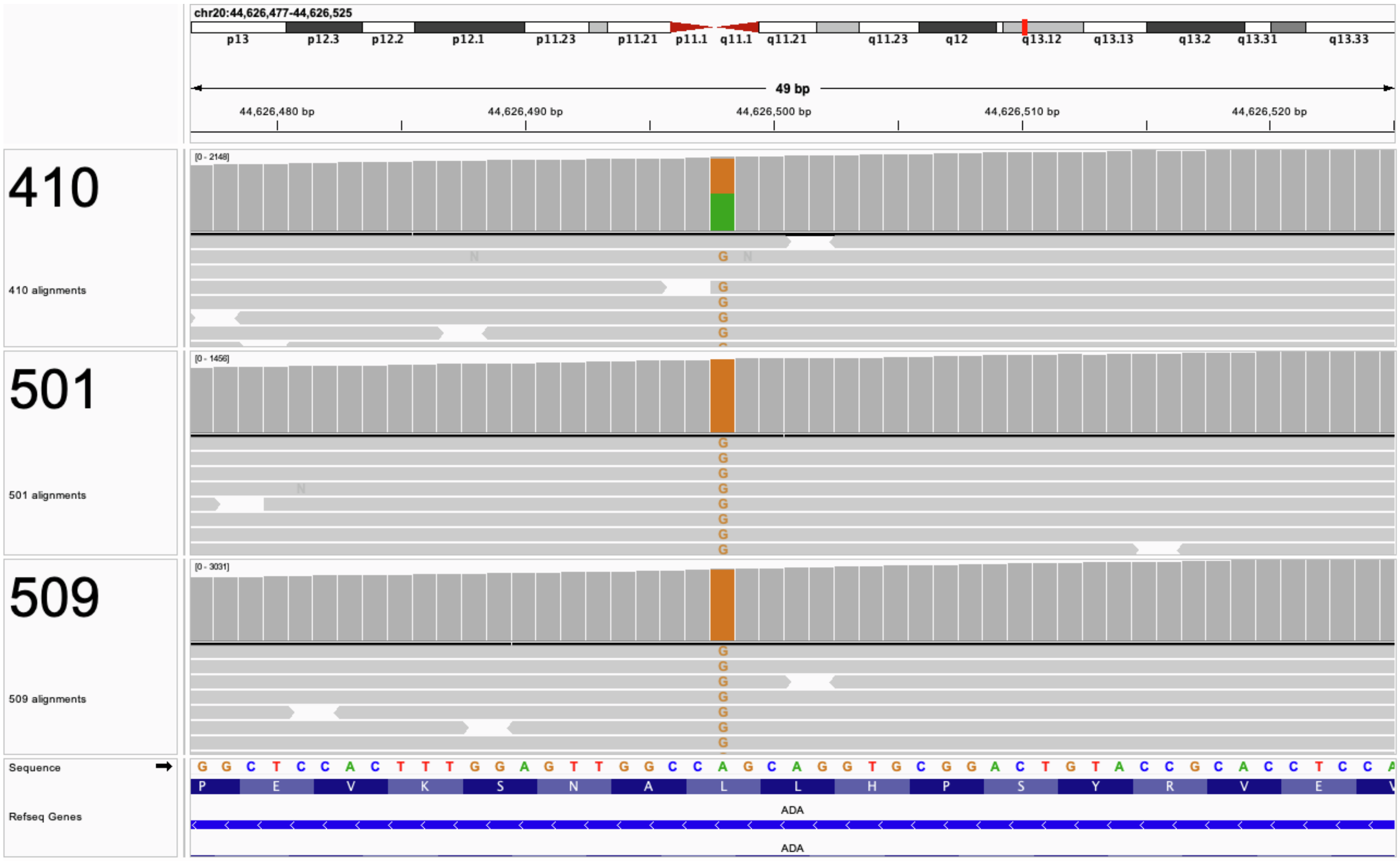

Figure S7: Visualization of Patients 410, 501, and 509 ADA-SCID Mutations (L107P)
Integrative genomics viewer visualization of ADA-SCID mutations.

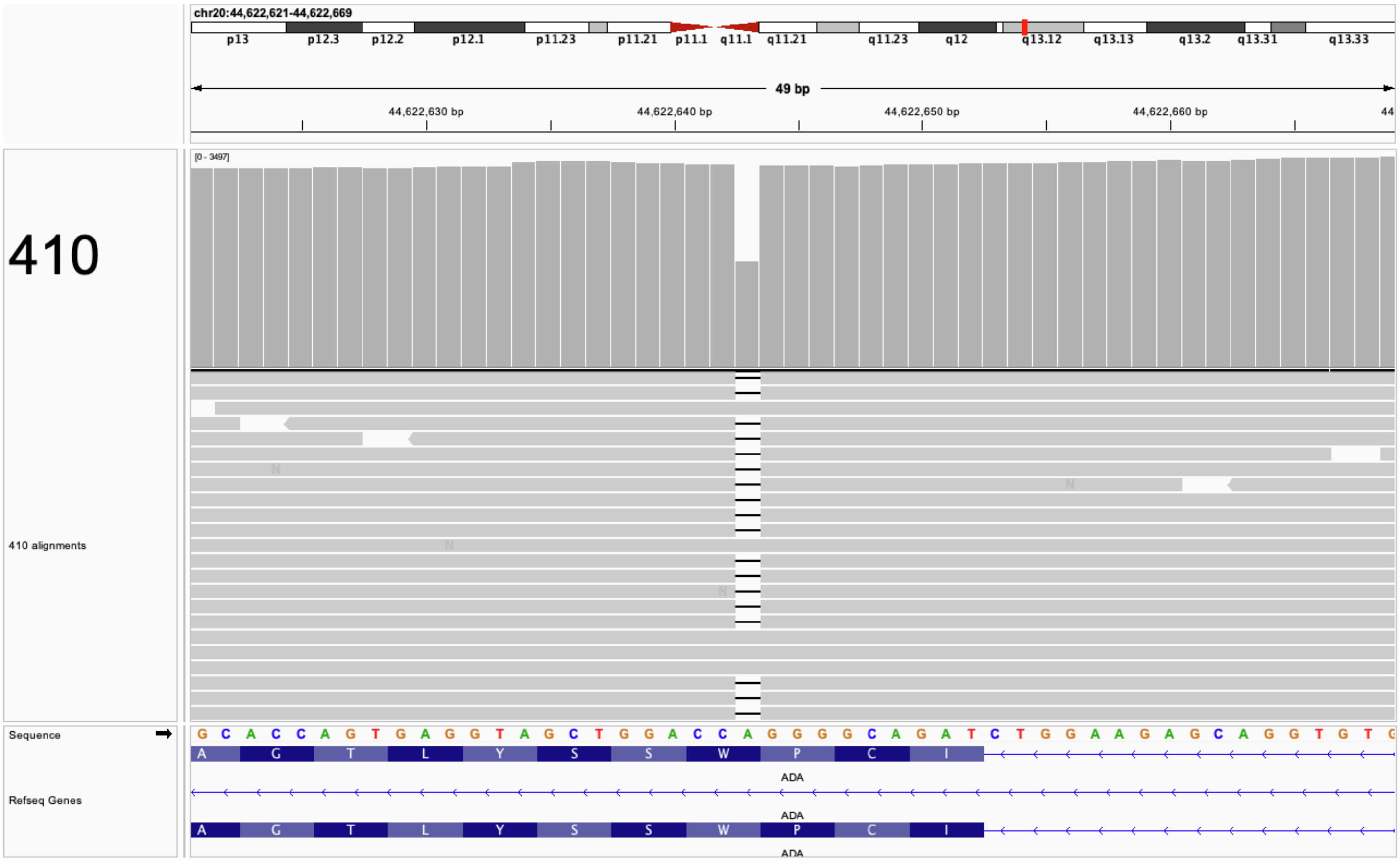

Figure S6: Visualization of Patients 410 ADA-SCID Mutation (c.790delT)
Integrative genomics viewer visualization of ADA-SCID mutations.

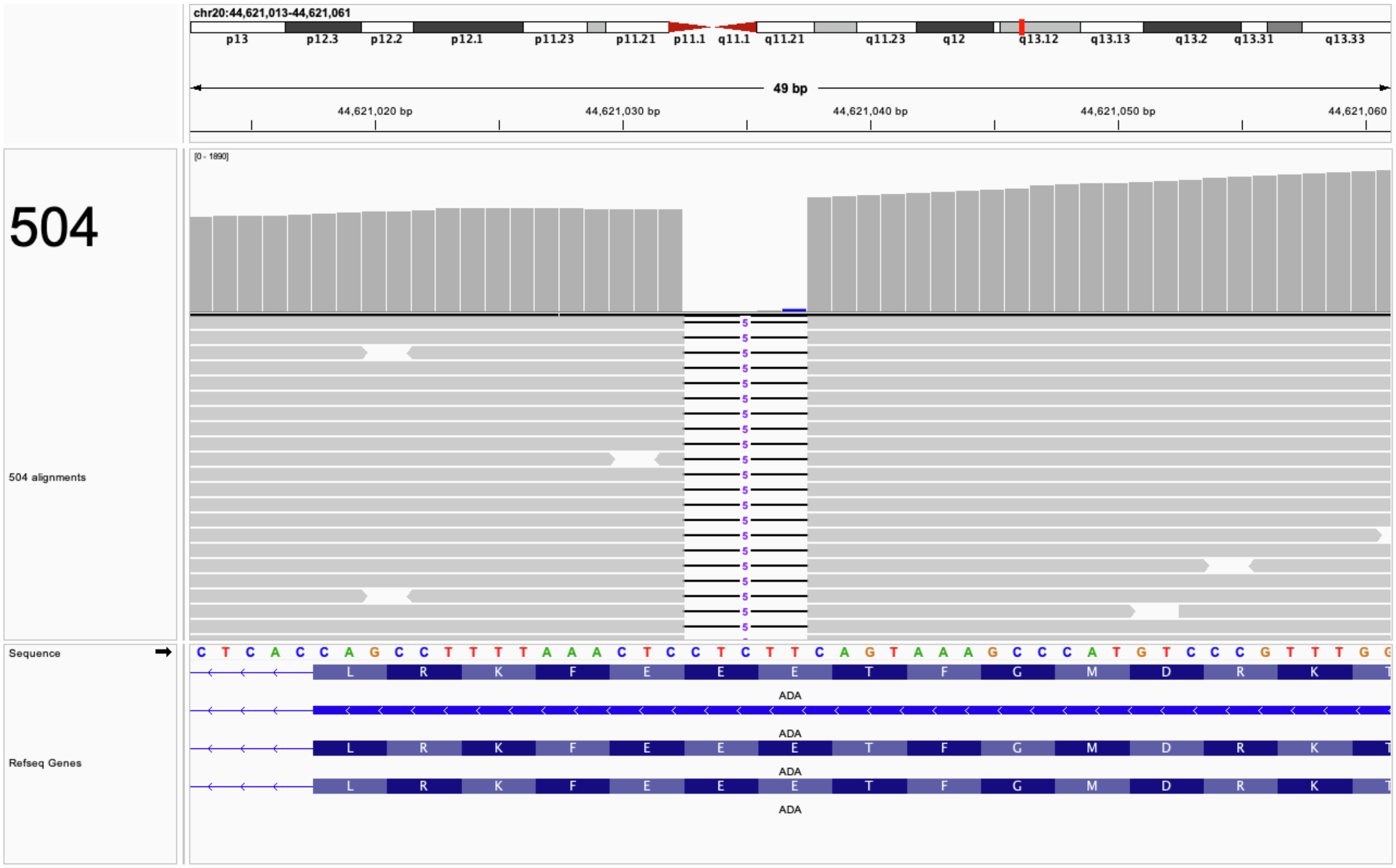

Figure S8: Visualization of Patient 504 ADA-SCID Mutations (G216R)
Integrative genomics viewer visualization of ADA-SCID mutations.

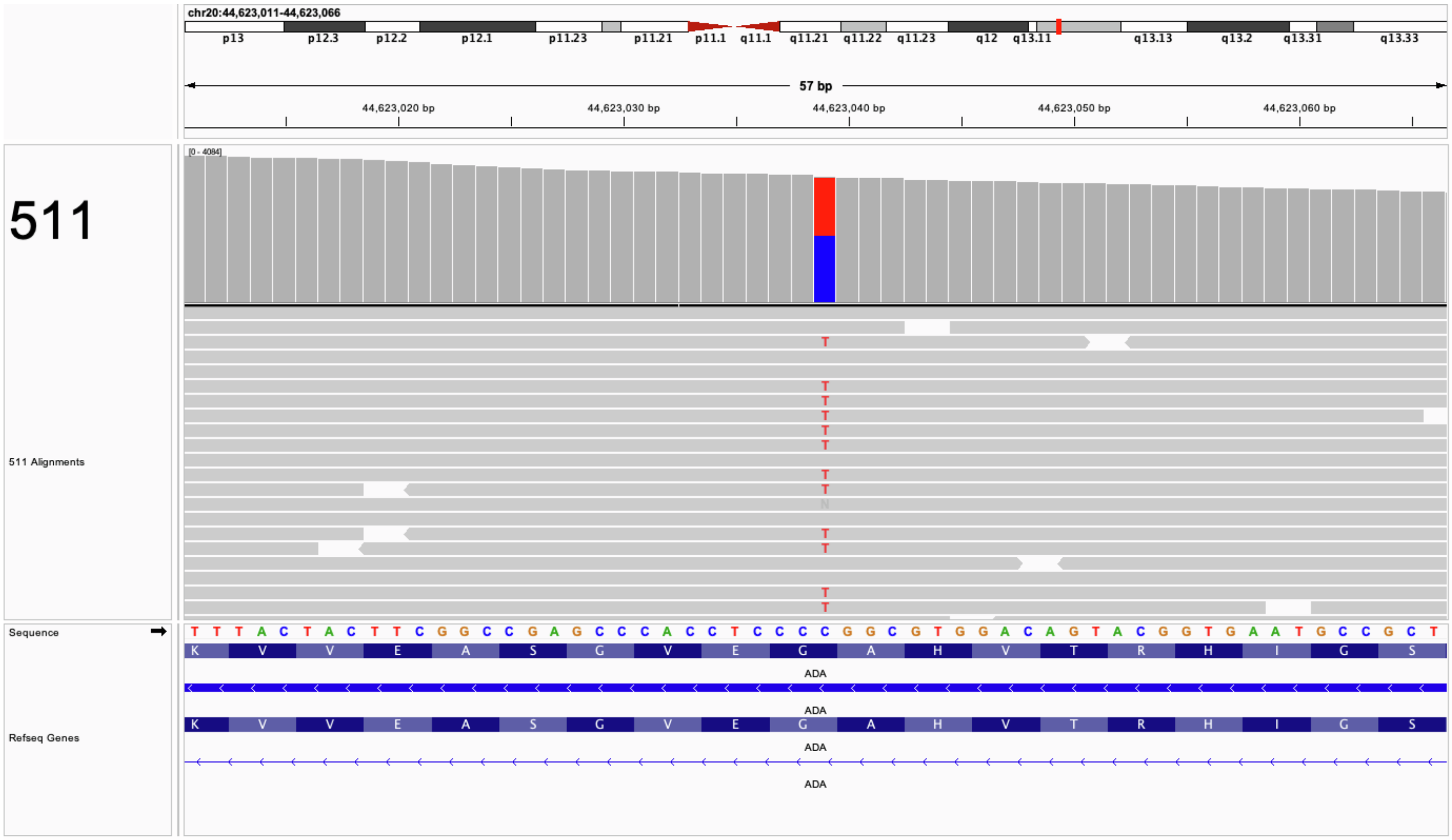

Figure S9: Visualization of Patient 511 ADA-SCID Mutation (G216R)
Integrative genomics viewer visualization of ADA-SCID mutations.

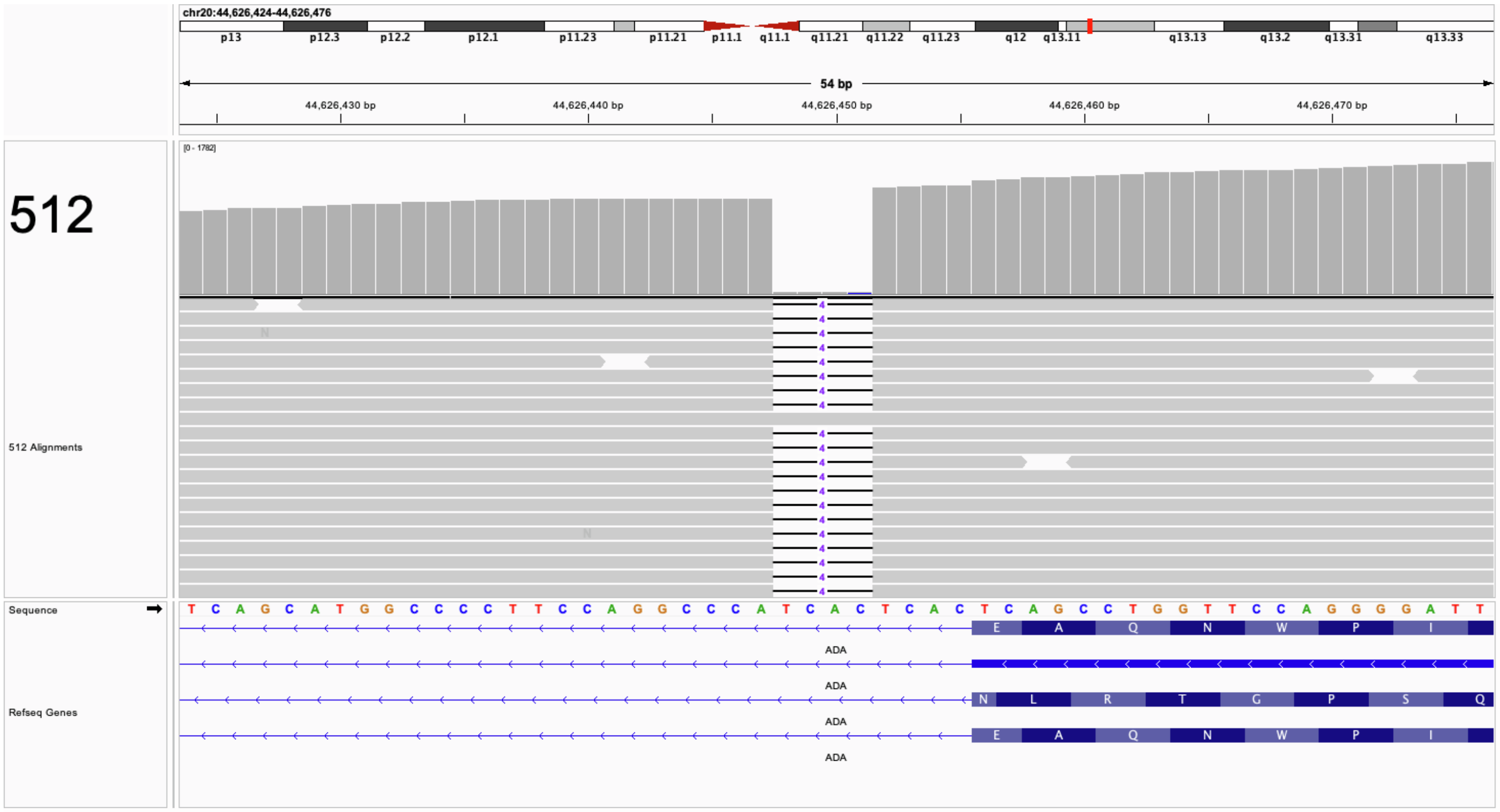

Figure S10: Visualization of Patient 512 ADA-SCID Mutations (c.360-362+1delTGAG)
Integrative genomics viewer visualization of ADA-SCID mutations.

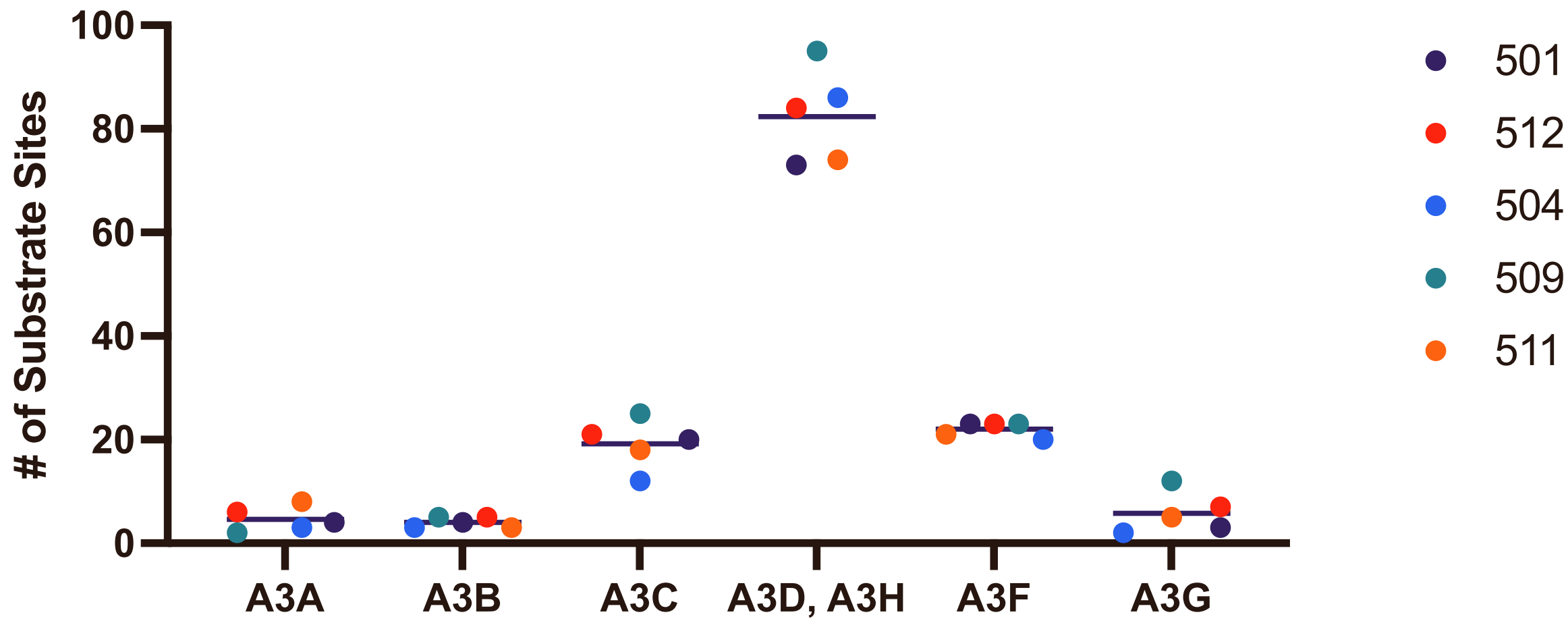

Figure S11: APOBEC3 Family Protein Prediction in PBMCs
APOBEC3 substrate sites were analyzed to assess the DNA sequence at the mutation sites. A3D and A3H have the same substrate site and same number of calls so were grouped together.

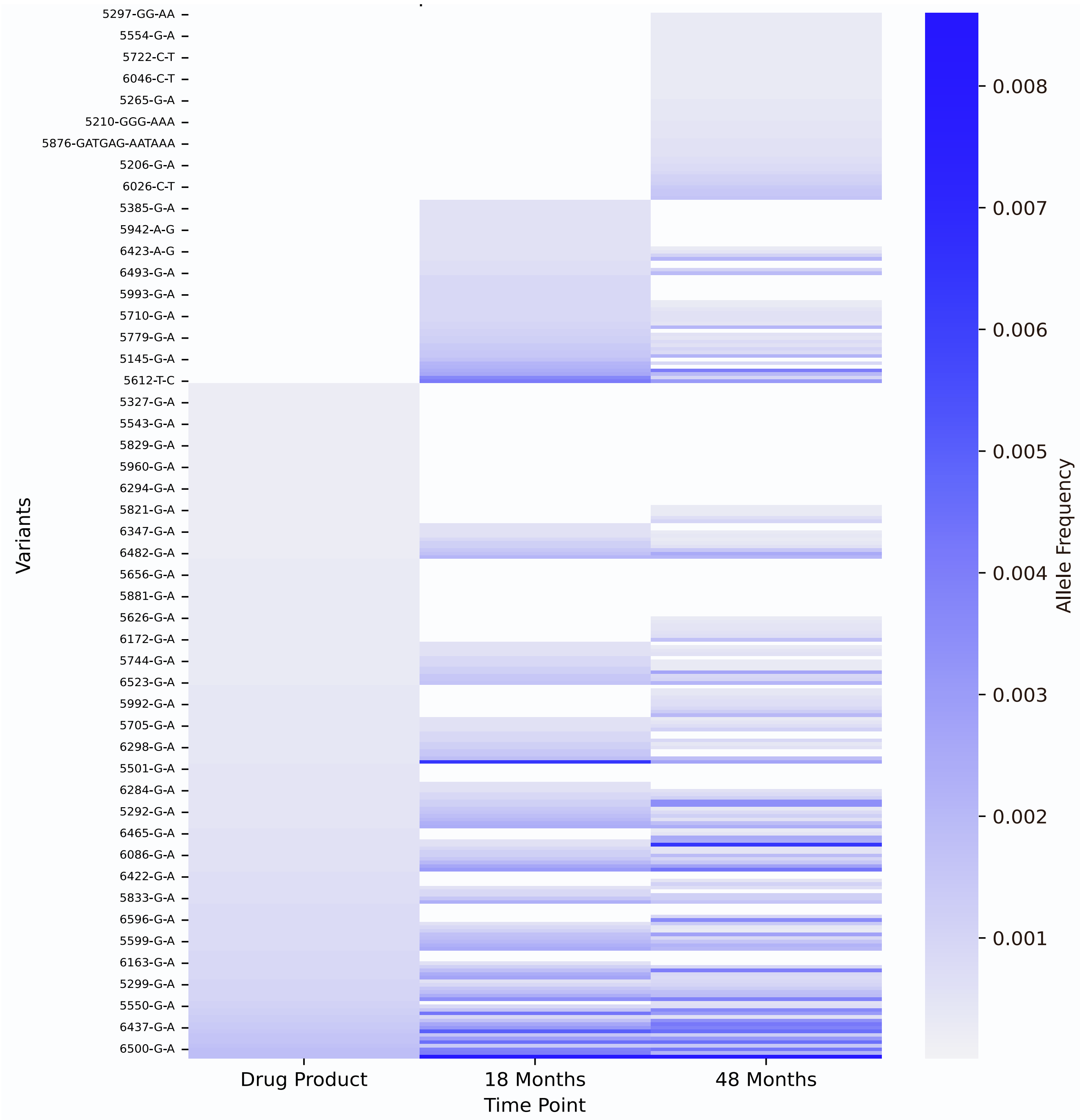

Figure S12: Heatmap of 607 Variant Allele Frequency Over Time
Allele frequencies were compared from each variant in the retention sample of the drug product with the variants of the PBMC DNA samples post gene therapy. frequencies = 0.000 are shown in white indicating that variant was not called at that time point. For visual clarity, only a subset of y-axis labels is displayed.

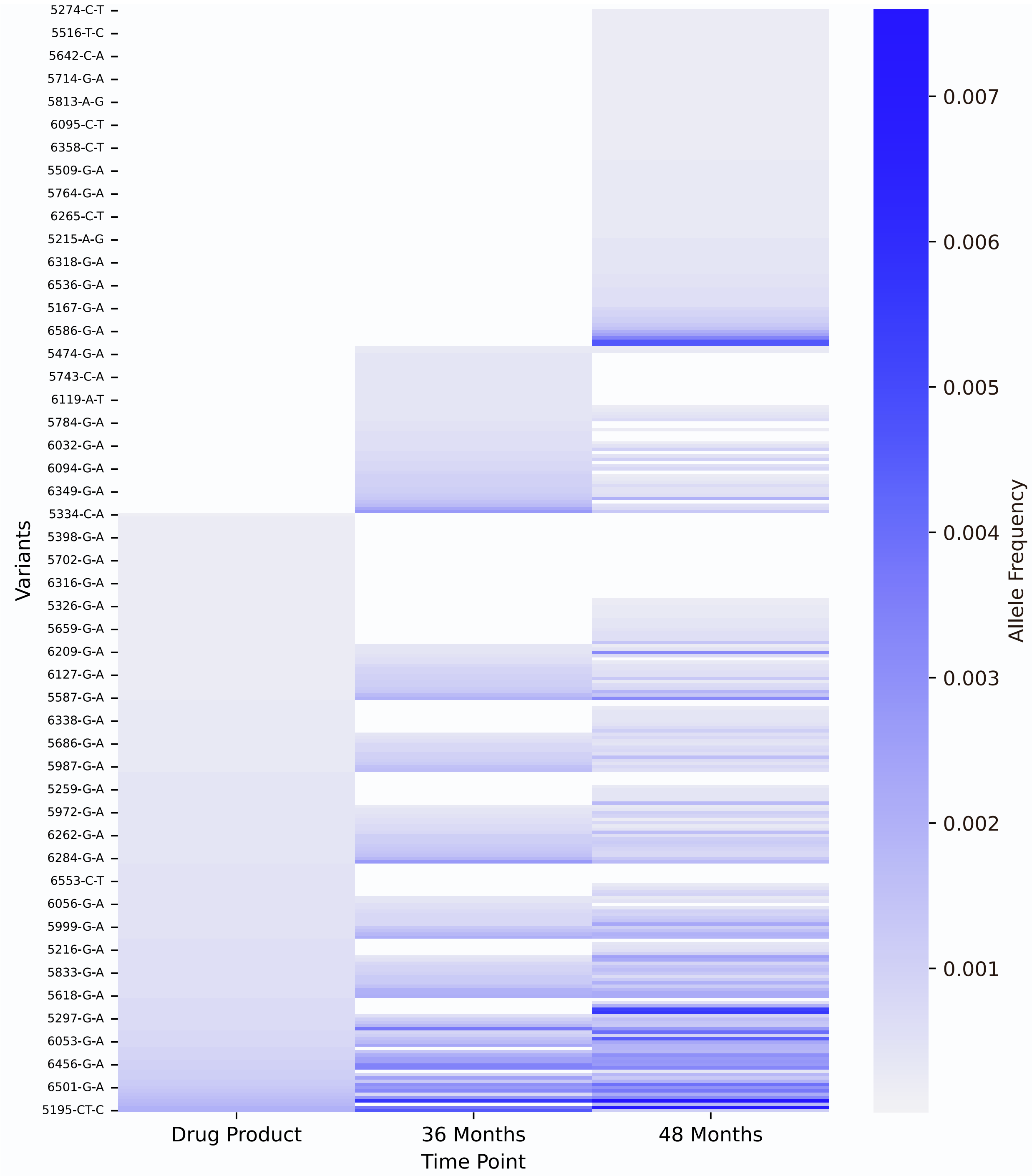

Figure S13: Heatmap of 609 Variant Allele Frequency Over Time
Allele frequencies were compared from each variant in the retention sample of the drug product with the variants of the T-Cell DNA sample post gene therapy. Allele frequencies = 0.000 are shown in white indicating that variant was not called at that time point. For visual clarity, only a subset of y-axis labels is displayed.

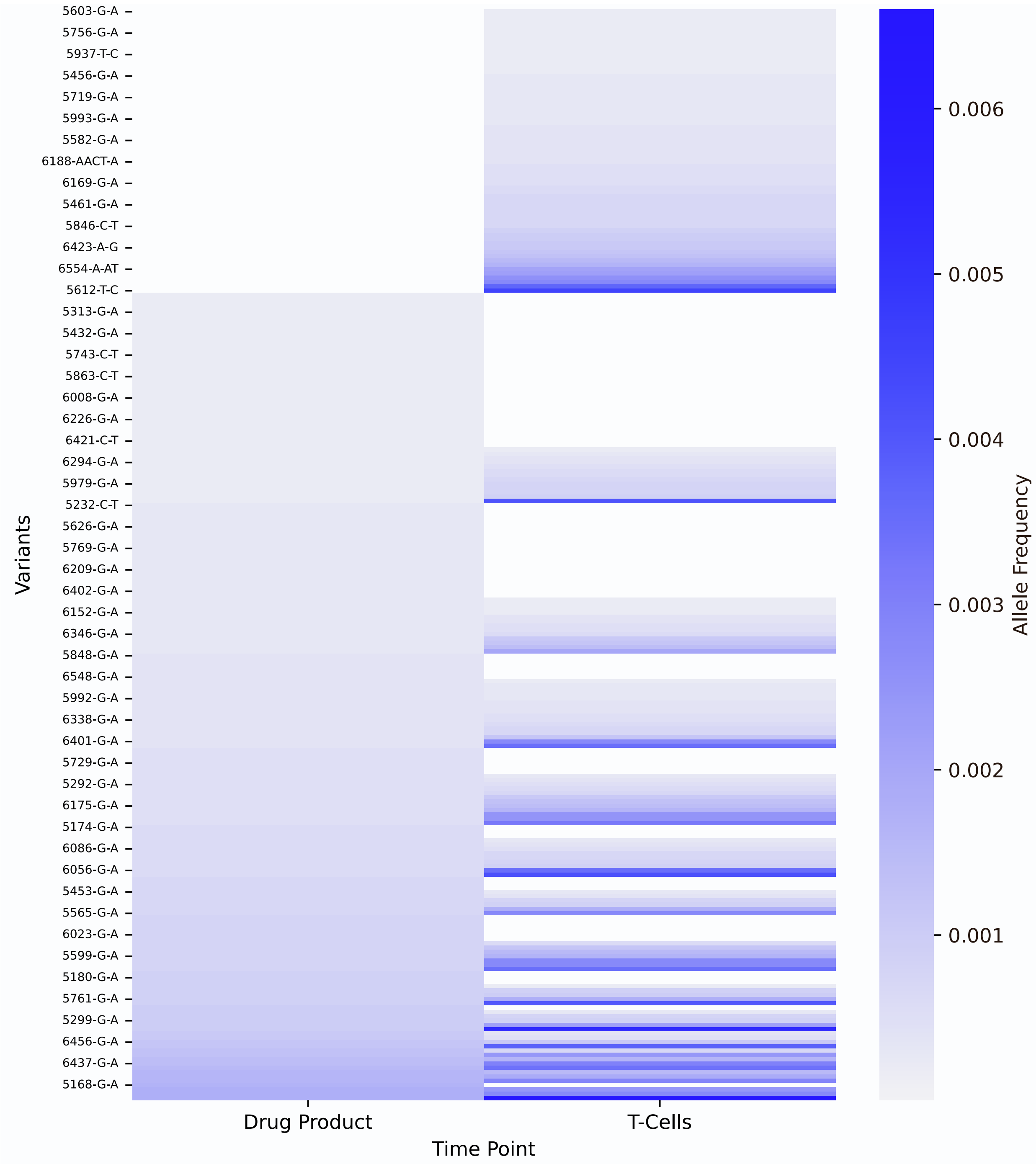

Figure S14: Heatmap of 607 Drug Product Variants vs. T-Cell Variants
Allele frequencies were compared from each variant in the retention sample of the drug product with the variants of the PBMC DNA samples post gene therapy. Allele frequencies = 0.000 are shown in white indicating that variant was not called at that time point. For visual clarity, only a subset of y-axis labels is displayed.

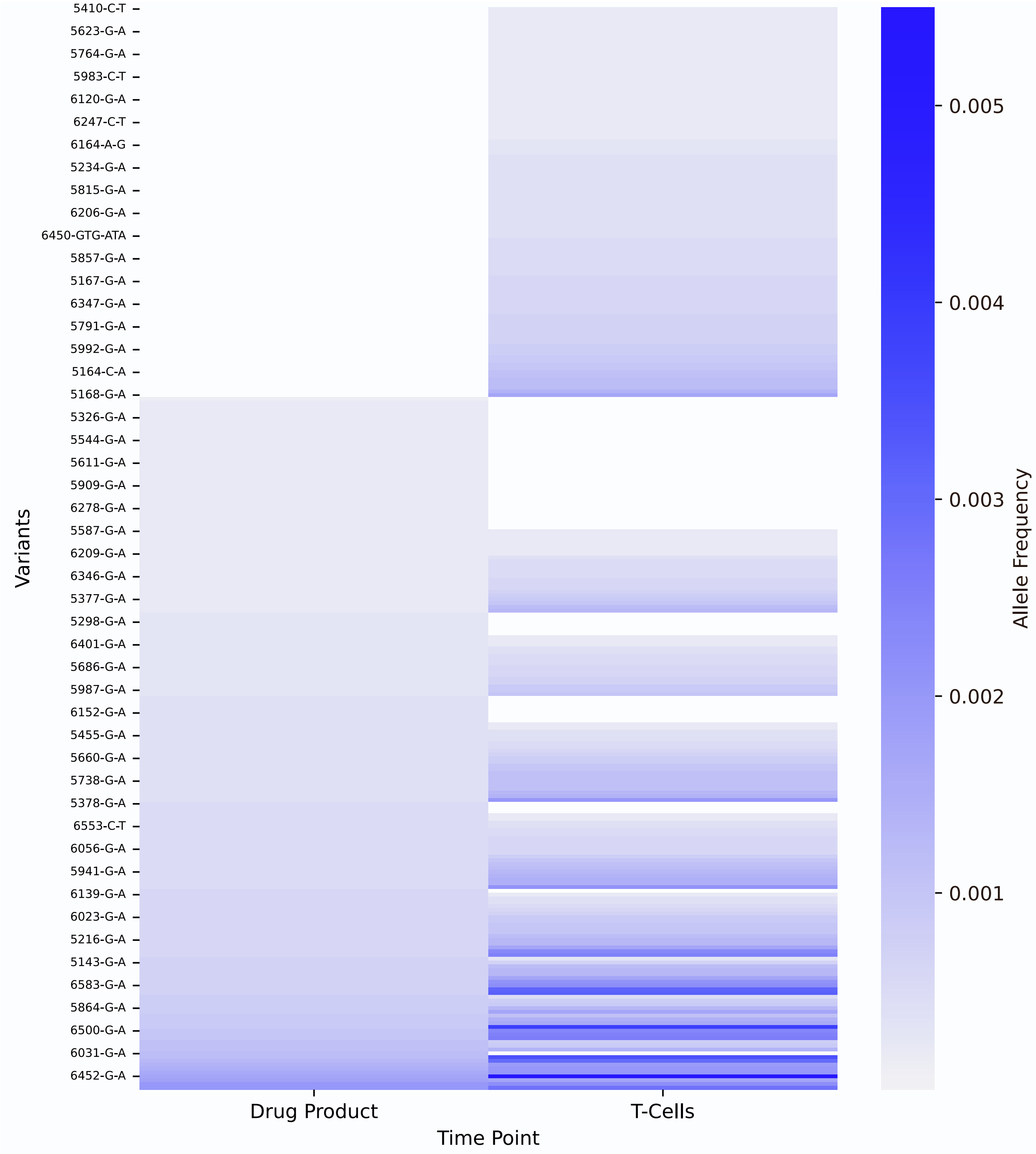

Figure S15: Heatmap of 609 Drug Product Variants vs. T-Cell Variants

Allele frequencies were compared from each variant in the retention sample of the drug product with the variants of the T-Cell DNA sample post gene therapy. Allele frequencies = 0.000 are shown in white indicating that variant was not called at that time point. For visual clarity, only a subset of y-axis labels is displayed.

Table S1: Transduced CD3+ DNA Sequencing Statistics

Sample #	Vector	Capture	Data (Gb)	Read Total	Total Primary Alignments	Total Alignments to Transgene	Total Alignments to Endogenous *ADA*	% On Target Alignments	ADA cDNA Mean Coverage
Sequencing Statistics Pre Error-Correction, Read Collapsing, and Filtering									
2	MND-ADA (VCN: 0.7)	Single	42.99	863,334,033	862,560,476	88,601,959	173,968,486	30.44%	9.89e+05x
1	EFS-ADA (VCN: 3)	Single	41.77	838,838,523	838,166,931	197,672,540	150,835,681	41.58%	9.92e+05x
4	MND-ADA (VCN: 0.7)	Double	10.38	207,812,221	207,656,042	78,438,755	11,413,916	43.26%	9.78e+05x
3	EFS-ADA (VCN: 3)	Double	72.35	1,448,376,160	1,447,352,878	1,247,407,423	181,196,855	98.70%	9.92e+05x
Sequencing Statistics Post Error Correction, Read Collapsing, and Filtering									
2	MND-ADA (VCN: 0.7)	Single	0.22	314,729	314,650	74,371	223,273	94.60%	3.53e+03x
1	EFS-ADA (VCN: 3)	Single	0.32	439,440	439,439	231,324	195,670	97.17%	1.77e+04x
4	MND-ADA (VCN: 0.7)	Double	0.21	360,895	360,871	90,609	254,405	95.61%	4.42e+03x
3	EFS-ADA (VCN: 3)	Double	0.58	718,074	718,063	370,100	326,895	97.07%	2.72e+04x

CD3+ cells were transduced with either γ-retrovirus (MND-ADA) or lentivirus (EFS-ADA) and sequenced. A single hybridization capture versus a double hybridization capture was performed. Sequencing reads were aligned to the human genome (hg38) and either the MND-ADA or EFS-ADA vector sequence pre and post error-correction, read collapsing, and filtering. Percent on target alignments was calculated as total alignments to transgene added to total alignments to endogenous *ADA* divided by the total alignments. Mean depth coverage was calculated from the start to end position of the ADA cDNA.

Table S2: Transduced CD3+ Cell Transgene Variants (Single vs. Double Hybridization Capture Comparison)

	**γ-Retrovirus (MND-ADA)**		**Lentivirus (EFS-ADA)**	
**Sample #**	2	4	1	3
**Capture**	Single	Double	Single	Double
**Total Mutations**	2	1	53	72
**Purine to Purine**				
A to G	0	0	0	0
G to A	0	0	48	59
**Pyrimidine to Pyrimidine**				
C to T	1	1	0	1
T to C	1	0	1	2
**Purine to Pyrimidine**				
A to C	0	0	1	0
A to T	0	0	0	0
G to C	0	0	0	0
G to T	0	0	0	0
**Pyrimidine to Purine**				
C to A	0	0	0	0
C to G	0	0	0	1
T to A	0	0	0	0
T to G	0	0	0	0
**Deletion/Insertion**	0	0	1	6
**Complex**	0	0	2	3

DNA Mutation profile for CD3+ cells transduced with γ-retrovirus (VCN=0.7) and lentivirus (VCN=3). VarDictJava was utilized to identify variants (allele frequency = 0.001).

Table S3: Gene Therapy Patient Demographics

Patient ID	Years Post Gene Therapy	ADA-SCID Mutations	VCN (Granulocytes)	VCN (PBMCs)
402 (γ-RV)	10	T+2>G in IVS7 / unknown-unidentified	0.0167	0.512
404 (γ-RV)	4.5	T+2>G in IVS7 / unknown-unidentified	0.0956	0.145
405 (γ-RV)	5	R211H / R211H	0.0406	0.474
408 (γ-RV)	7	R211H / R211H	0.183	1.29
410 (γ-RV)	6	L107P / c.790delT	0.204	0.91
501 (LVV)	6	L107P / L107P	0.466	2.02
504 (LVV)	8	delc.955-959 / delc.955-959	0.238	0.625
509 (LVV)	8	L107P / L107P	0.399	1.594
511 (LVV)	8	G216R / c.(Exon1)del	0.751	1.99
512 (LVV)	5	c.360-362+1delTGAG / c.360-362+1delTGAG	0.149	1.59

(γ-RV) = MND-ADA gamma-retroviral vector

(LVV) = EFS-ADA lentiviral vector

Table S4: Information on CD3+ T-Cell Variants Identified in the Endogenous *ADA* gene locus

Variant	Position	Region	Allele	Allele Frequency (Our Dataset)	Validated Frequency (Global Study)	Consequences
SNV	Chr20:44636867	Intron 1	A>G,T	1	G=0.9719 (1000Genomies30x)	Benign
SNV	Chr20:44636286	Exon 2	C>A,**T**	1	T=0.99286 (ALFA) T=0.9816 (1000Genomies30x)	Synonymous, Benign
SNV	Chr20:44636062	Intron 2	T>A,C,G	0.5088	C=0.89038 (ALFA) C=0.9197 (1000Genomies30x)	Benign
SNV	Chr20:44636050	Intron 2	C>G,T	0.511	G=0.6966 (1000Genomies30x)	Benign
SNV	Chr20:44628564	Intron 3	G>T	0.2857	T=0.000007 (gnomAD) T=0.000011 (TopMED)	Benign
SNV	Chr20:44626054	Intron 3	G>T	0.2023	T=0.07045 (ALFA) T=0.1138 (1000Genomies30x)	Benign
SNV	Chr20:44624853	Intron 5	C>T	0.1875	T=0.20628 (ALFA) T=0.2631 (1000Genomies30x)	Benign
SNV	Chr20:44624274	Exon 6	T>C	0.188	C=0.185627 (ALFA) C=0.2728 (1000Genomies30x)	Synonymous, Benign
SNV	Chr20:44623126	Intron 6	G>A	0.1603	A=0.12629 (ALFA) A=0.1668 (1000Genomies30x)	Benign
SNV	Chr20:44622793	Intron 8	C>T	0.1744	A=0.12629 (ALFA) A=0.1668 (1000Genomies30x)	Benign
SNV	Chr20:44622081	Intron 9	G>A	0.15	A=0.162783 (ALFA) A=0.2616 (1000Genomes30x)	Benign
SNV	Chr20:44620950	Intron 9	C>T	0.1711	T=0.063452 (ALFA) T=0.1106 (1000Genomes30x)	Benign

Mutations in the endogenous *ADA* gene that are identified in our analysis are recognized as common variants found in the Single Nucleotide Polymorphism (SNP) database. Validated frequency from two common genome sequencing datasets, Allele Frequency Aggregator (ALFA) dataset and 1000 Genomes Project Phase 3 (1000Genomes_30x), are shown. Mutations that are also identified in our analysis are underlined. Consequences are classified in the Reference SNP Reports of dbSNP database. Benign variants are reported on ClinVar database.

Table S5: Gene Therapy Patient Sequencing Statistics Pre Error-Correction, Read Collapsing and Filtering

Sample #	Cell Type	Read Total	Total Primary Alignments	Total Alignments to Transgene	Total Alignments to Endogenous *ADA*	% On Target Alignments	ADA cDNA Mean Coverage
402	Granulocytes	733,080,304	731,926,939	822,451	28,242,229	3.97%	7.86+04x
402	PBMCs	842,459,801	841,678,430	7,389,807	73,381,043	9.60%	7.76+05x
404	Granulocytes	740,752,536	739,745,435	823,965	285,076,681	38.68%	8.30+04x
404	PBMCs	727,564,741	726,824,794	4,636,516	61,429,158	9.09%	4.89+05x
405	Granulocytes	801,851,736	800,880,869	2,079,981	31,738,329	4.2%	2.16+05x
405	PBMCs	883,139,674	879,659,663	9,243,470	81,099,137	10.3%	7.15+05x
408	Granulocytes	1,143,708,875	1,141,987,552	2,132,258	3,772,878	0.52%	2.20+05x
408	PBMCs	1,068,953,540	1,067,809,656	57,102,608	98,006,418	14.53%	9.92+05x
410	Granulocytes	635,950,786	634,658,951	2,555,917	26,240,126	4.5%	2.65+05x
410	PBMCs	969,919,482	965,932,333	21,636,634	82,880,816	10.82%	9.82+05x
501	Granulocytes	729,781,985	728,761,412	10,137,490	36,318,448	6.37%	9.62+05x
501	PBMCs	430,722,037	430,374,338	25,480,720	20,226,687	10.62%	9.92+05x
504	Granulocytes	736,640,959	735,283,398	4,646,323	39,505,055	6.00%	4.96+05x
504	PBMCs	597,588,757	596,972,362	12,018,633	28,964,866	6.87%	9.83+05x
509	Granulocytes	660,283,113	659,524,628	10,961,293	33,728,858	6.78%	9.77+05x
509	PBMCs	602,150,898	601,595,794	27,632,462	32,269,522	9.96%	9.92+05x
511	Granulocytes	609,124,973	608,352,580	12,982,860	29,753,015	7.02%	9.86+05x
511	PBMCs	439,207,503	438,873,190	26,806,684	21,046,498	10.90%	9.92+05x
512	Granulocytes	596,660,053	597,076,342	3,666,009	30,996,413	5.81%	3.93+05x
512	PBMCs	544,025,568	544,025,568	22,120,139	25,590,784	8.77%	9.92+05x

Table S6: Gene Therapy Patient Sequencing Statistics Post Error-Correction, Read Collapsing and Filtering

Sample #	Cell Type	Read Total	Total Primary Alignments	Total Alignments to Transgene	Total Alignments to Endogenous *ADA*	% On Target Alignments	ADA cDNA Mean Coverage
402	Granulocytes	56,314	56,268	1,254	53,256	96.88%	101.58x
402	PBMCs	69,446	69,444	4,290	62,004	95.46%	320.20x
404	Granulocytes	51620	51,607	1,330	48,853	97.24%	115.81x
404	PBMCs	55,406	55,394	4,031	50,012	97.56%	352.82x
405	Granulocytes	61,771	61,768	4,067	56,220	97.60%	366.18x
405	PBMCs	76,497	76,222	7,560	66,223	96.80%	624.68x
408	Granulocytes	74,244	74,231	2,614	67,655	94.66%	204.73x
408	PBMCs	101,772	101,772	28,359	69,674	96.32%	2.17+03x
410	Granulocytes	56,043	56,015	4,979	49,502	97.26%	424.67x
410	PBMCs	90,643	90,294	18,551	68,643	96.57%	1.55+03x
501	Granulocytes	57,150	57,143	14,433	41,921	98.92%	1.34+03x
501	PBMCs	110,374	110,374	64,128	45,408	99.24%	5.94+03x
504	Granulocytes	72,542	72,542	5,593	64,832	96.46%	461.53x
504	PBMCs	97,667	97,667	20,587	74,193	97.04%	1.66+03x
509	Granulocytes	69,749	69,749	12,578	55,272	97.28%	1.06+03x
509	PBMCs	121,305	121,305	42,111	76,526	97.8%	3.44+03x
511	Granulocytes	53,043	53,042	19,076	33,279	98.7%	1.75+03x
511	PBMCs	115,306	115,304	68, 709	45,715	99.24%	6.35+03x
512	Granulocytes	39,125	39,123	4,857	33,648	98.42%	446.87x
512	PBMCs	104,009	103,994	50,068	52,815	98.93%	4.51+03x

Table S7: Allele Frequencies of G to A Mutations in Lentiviral Treated Patient Cells

Table S8: Gene Therapy Patient Variants in the Endogenous *ADA* gene locus

	**γ-Retrovirus (MND-ADA)**										**Lentivirus (EFS-ADA)**									
	**Granulocytes**					**PBMCs**					**Granulocytes**					**PBMCs**				
**Sample**	**402**	**404**	**405**	**408**	**410**	**402**	**404**	**405**	**408**	**410**	**501**	**504**	**509**	**511**	**512**	**501**	**504**	**509**	**511**	**512**
**Total Mutations**	5	6	5	11	6	7	5	6	9	7	9	12	7	4	6	5	15	5	9	12
**Purine to Purine**																				
A to G	0	0	0	0	1	1	0	1	0	2	1	1	2	0	0	1	1	1	0	1
G to A	0	0	0	1	0	0	0	0	1	0	1	3	0	0	0	0	4	0	1	2
**Pyrimidine to Pyrimidine**																				
C to T	1	1	2	5	1	1	1	2	3	1	2	2	1	1	1	1	3	1	3	2
T to C	1	1	1	2	2	1	1	1	2	2	1	3	2	1	1	2	3	2	2	1
**Purine to Pyrimidine**																				
A to C	1	1	0	0	0	1	1	0	0	0	0	0	0	0	0	0	0	0	0	0
A to T	0	0	0	0	0	0	0	0	0	0	0	0	0	0	0	0	0	0	0	0
G to C	1	1	0	0	0	1	1	0	0	0	0	0	0	0	0	0	0	0	0	0
G to T	0	0	0	0	0	0	0	0	0	0	0	0	0	0	0	0	0	0	0	0
**Pyrimidine to Purine**																				
C to A	0	0	0	0	0	0	0	0	0	0	0	0	0	0	0	0	0	0	0	0
C to G	1	1	1	1	1	1	1	1	1	1	1	0	1	1	1	1	0	1	1	1
T to A	0	0	0	0	0	0	0	0	0	0	0	0	0	0	0	0	0	0	0	0
T to G	0	0	0	0	0	0	0	0	0	0	0	0	0	0	0	0	0	0	0	0
**Deletion/ Insertion**	0	0	1	2	1	1	0	1	2	1	2	3	1	0	3	1	4	0	3	5
**Complex**	0	1	0	0	0	0	0	0	0	0	0	0	0	0	0	0	0	0	0	0

ADA-SCID gene therapy patient PBMCs and granulocyte DNA was sequenced. VarDict was used to call variants at an allele frequency at 0.001. Patient identification numbers in the 400s received γ-retroviral gene therapy; those in the 500s received lentiviral vector gene therapy. Patient identification numbers in the 500s received lentiviral gene therapy.

Table S9: Identified Variants in the Endogenous *ADA* gene locus of patient samples; in database

Variant	Position	Region	Allele	Patient ID	Allele Frequency (Our Dataset)	Validated Frequency (Global Study)	Consequences
SNV	Chr20:44636867	Intron 1	A>G,T	402, 405, 410, 509, 512	1, 1, 1, 1,1	G=0.9719 (1000Genomes_30x)	Intron Variant
SNV	Chr20:44636580	intron 1	T>C	410, 501, 509	0.5352, 0.4889, 1	C=0.00173 (ALFA) C=0.0003 (1000Genomes_30x)	Intron Variant
SNV	Chr20:44636286	Exon 2	C>A,T	ALL	1, 1, 1, 1, 1, 1, 1, 1, 1, 1	T=0.99286 (ALFA) T=0.9816 (1000Genomes_30x)	Synonymous, Benign
SNV	Chr20:44636062	Intron 2	T>A,C,G	402, 404, 405, 408, 410, 501, 509, 511, 512	1, 1, 1, 1,1, 1, 1, 1, 1	C=0.89038 (ALFA) C=0.9197 (1000Genomes_30x)	Intron Variant
SNV	Chr20:44636050	Intron 2	C>G,T	402, 404, 405, 408, 410, 501, 509, 511, 512	1, 1, 1, 1, 1, 05322, 1, 1, 1	G=0.6583 (ALFA) G=0.6966 (1000Genomes_30x)	Intron Variant
SNV	Chr20:44629073	Exon 3	C>T	501, 511, 512	0.0096(G), 0.0033, 0.0262	T=0.0006 (1000Genomes_30x)	Synonymous, Benign
SNV	Chr20:44628765	Intron 3	C>T	504	0.9863	T=0.068580 (ALFA) T=0.0595 (1000Genomes_30x)	Intron Variant
SNV	Chr20:44626579	Exon 4	T>C	504	0.9995	C=0.063659 (ALFA) C=0.0517 (1000Genomes_30x)	K80R Missense Variant, Benign
**SNV**	**Chr20:44626498**	**Exon 4**	**A>G**	**410, 501, 509**	**0.4848, 1, 1**	**G=11 (ClinVar)**	**L107P Missense Pathogenic**
SNV	Chr20:44625657	Exon 5	C>T	408	0.4998	T=0.03738 (ALFA) T=0.0323 (1000Genomes_30x)	Synonymous
SNV	Chr20:44624853	Intron 5	C>T	408, 504	1, 1	T=0.20628 (ALFA) T=0.2631 (1000Genomes_30x)	Intron Variant
**SNV**	**Chr20:44624292**	**Exon 6**	**G>C**	**402, 404**	**0.4781, 0.4522**	**GTA > CTA (Analysis) GTA>TTA (ClinVar)**	**Y>stop, Y>stop, Pathogenic**
SNV	Chr20:44624274	Exon 6	T>C	408, 504	0.505, 1	C=0.185627 (ALFA) C=0.2728 (1000Genomes_30x)	Synonymous, Benign
SNV	Chr20:44624007	Intron 6	A>G	504	0.9944	G=0.07464 (ALFA) G=0.0623 (1000Genomes_30x)	Intron Variant
SNV	Chr20:44623126	Intron 6	G>A	504	0.9992	A=0.12629 (ALFA) A=0.1668 (1000Genomes_30x)	Intron Variant
**SNV**	**Chr20:44623053**	**Exon 7**	**C>T**	**405, 408**	**0.9995, 1**	**ACG>ATG T=11 (ClinVar)**	**R211H Missense, Pathogenic**
**SNV**	**Chr20:44623039**	**Exon 7**	**C>T**	**511**	**0.4696**	**T=0.00011 (ALFA) T=13 (ClinVar)**	**G216R Missense, Pathogenic**
SNV	Chr20:44623005	Intron 7	A>C	402, 404	0.4858, 0.4822	C=0.000007 (gnomAD)	Splice Donor Variant
SNV	Chr20:44622793	Intron 8	C>T	408	0.49	A=0.12629 (ALFA) A=0.1668 (1000Genomes_30x)	Intron Variant
**DEL**	**Chr20:44622642**	**Exon 9**	**CA>C**	**410**	**0.4946**	**delA=3 (ClinVar)**	**Frameshift, Likely-Pathogenic**
SNV	Chr20:44622081	Intron 9	G>A	408, 504	0.4706, 1	A=0.162783 (ALFA) A=0.2616 (1000Genomes_30x)	Intron Variant
**DEL**	**Chr20:44621032**	**Exon 10**	**CCTCTT>C**	**504**	**0.9984**	**Deletion of E319-320**	**Frameshift**
SNV	Chr20:44620212	Intron 11	T>C	504	1	C=0.06501 (ALFA) C=0.0754 (100Genomes_30x)	Intron Variant
SNV	Chr20:44620200	Intron 11	G>A	504	1	A=0.07009 (ALFA) A=0.0732 (1000Genomes_30x)	Intron Variant

Mutations in the endogenous *ADA* gene that are identified in our analysis are recognized as common variants found in the Single Nucleotide Polymorphism (SNP) database. Validated frequency from two common genome sequencing datasets, Allele Frequency Aggregator (ALFA) dataset and 1000 Genomes Project Phase 3 (1000Genomes_30x), NHBLI's Trans-Omics for Precision Medicine Whole Genome Sequencing Project (TopMed), Genome Aggregation Database (gnomAD), are shown. For pathogenic mutations, the number of submissions on ClinVar are shown. Mutations that are also identified in our analysis are underlined. Consequences are classified in the Reference SNP Reports of dbSNP database. Benign and Pathogenic variants are reported on ClinVar database. Patient ID's are summarized for every identified variant. ADA-SCID mutations are in blue.

Table S10: Identified Variants in the Endogenous *ADA* gene locus of patient samples; but not in the current database

Variant	Position	Region	Allele	Patient ID	Allele Frequency (Our Dataset)	Potential consequences
DEL	Chr20:44636307	Intron 1	AAG>A	405, 504	0.002, 0.0019 (G)	Intron Variant
SNV	Chr20:44636307	Intron 1	G>A	408	1	Intron Variant
DEL	Chr20:44635902	Intron 2	G>GA	402, 405, 501, 504	0.0504, 0.0462, 0.381, 0.0435	Intron Variant
DEL	Chr20:44635902	Intron 2	GA>G	404, 405, 408, 501, 504	0.0366 (G), 0.0462 0.0495, 0.381, 0.0164	Intron Variant
INS	Chr20:44629417	Intron 2	A>AGT	408, 504, 509,	0.3351, 0.6364, 0.0103	Intron Variant
SNV	Chr20:44626545	Exon 4	G>A	501, 511, 512	0.0076, 0.0013, 0.0248	Synonymous, Benign
DEL	Chr20:44626447	Intron 4	ATCAC >A	512	0.9842	Intron Variant
DEL	Chr20:44625684	Exon 5	Large deletion	501, 511, 512	0.0119, 0.0055, 0.0455	Potentially Pathogenic
DEL	Chr20:44625291	Intron 5	AT>A	408	0.0208,	Intron Variant
DEL	Chr20:44622929	Exon 8	Large deletion	501, 512	0.0029 (G), 0.0065	Potentially Pathogenic
DEL	Chr20:44622652	Exon 9	Large deletion	501, 512	0.0028 (G), 0.0065	Potentially Pathogenic
SNV	Chr20:44621168	Intron 9	G>A	504, 512	0.0036, 0.0032	Intron Variant
DEL	Chr20:44620401	Exon 11	Large deletion	509, 512	0.0035, 0.0138	Potentially Pathogenic

Mutations in the endogenous ADA gene that are identified in our analysis but not recognized as common variants found in the Single Nucleotide Polymorphism (SNP) database. Patient ID’s are summarized for every identified variant.

Table S11: APOBEC3 DNA Substrate Statistics in EFS-ADA Treated Patients

	Granulocytes					PBMCs				
Sample	501	504	509	511	512	501	504	509	511	512
Total G to A Mutations	72	15	68	58	18	74	73	103	75	92
Mutations with APOBEC3 Substrate Signature	64	14	53	46	16	65	66	88	65	76
% APOBEC3 Signature	88.9%	93.3%	77.9%	79.3%	88.9%	87.8%	90.4%	85.4%	86.7%	82.6%

Table S12: Sequencing Statistics of CD34+ Cells Transduced with Viral Preparations from Parental and APOBEC3 Knock-out HEK293T cell line

Sample ID	Read Total	Total Primary Alignments	Total Alignments to Transgene	Total Alignments to Endogenous *ADA*	% On Target Alignments	ADA cDNA Mean Coverage
Sequencing Statistics Pre Error Correction, Read Collapsing, and Filtering						
Parental	875,460,830	874,497,489	89,043,481	84,058,771	19.79%	9.92e+05x
APOBEC3 KO	806,106,974	805,427,649	63,031,796	81,517,202	17.95%	9.92e+05x
Sequencing Statistics Post Error Correction, Read Collapsing, and Filtering						
Parental	487188	487188	214168	245789	94.41%	1.54e+04x
APOBEC3 KO	408950	408950	150068	233570	93.81%	1.10e+04x

Table S13: Gene Therapy Patient Drug Product, PMBC, and T-Cell Demographics

Patient ID	Sample Type	Months Post Gene Therapy	ADA-SCID Mutations	VCN
607	Drug Product	0	delc.956-960/ L107R	3.21
607	PBMC	18	delc.956-960/ L107R	0.56
607	PBMC	48	delc.956-960/ L107R	1.395
607	T-Cell	24	delc.956-960/ L107R	3.06
609	Drug Product	0	delc.956-960/ unidentified	2.97
609	PBMC	36	delc.956-960/ unidentified	2.18
609	PBMC	48	delc.956-960/ unidentified	1.74
609	T-Cell	24	delc.956-960/ unidentified	1.94

Table S14: Sequencing Statistics of Patient Drug Product, PBMCs, and T-cells

Sample	Read Total	Total Primary Alignments	Total Alignments to ADA Transgene	Total Alignments to Endogenous ADA	%On-Target Alignment (transgene)	%On-Target Alignment (Trans+Endogenous)	Transgene Mean Coverage
Sequencing Statistics Pre-Error Correction, Read Collapsing, and Filtering							
607 Drug Product	327291809	326186000	88886326	15583426	27.25%	32.03%	9.92E+05
607 PBMC 18 Months	241500931	240699458	9594272	46642464	3.99%	23.36%	9.07E+05
607 PBMCs 48 Months	235567288	234656353	18908719	37105180	8.06%	23.87%	9.92E+05
607 T-Cells	340300322	338912377	29266166	16980770	8.64%	13.65%	9.92E+05
609 Drug Product	368669061	367076608	129450471	14174362	35.27%	39.13%	9.92E+05
609 PBMC 36 Months	301173690	296914522	20041972	15371513	6.75%	11.93%	9.91E+05
609 PBMC 48 Months	889733534	881800024	25830039	39482889	2.93%	7.41%	9.92E+05
609_T-Cells	162064728	161696839	17695328	27253080	10.94%	27.80%	9.92E+05
Sequencing Statistics Post-Error Correction, Read Collapsing, and Filtering							
607 Drug Product	279801	279801	240886	38310	86.09%	99.78%	2.17E+04
607 PBMC 18 Months	339314	339314	46354	278872	13.66%	95.85%	3.36E+03
607 PBMC 48 Months	314630	314630	92326	210590	29.34%	96.28%	6.68E+03
607 T-Cells	121258	121258	83897	36843	69.19%	99.57%	7.94E+03
609 Drug Product	406062	406062	365759	39484	90.07%	99.80%	3.11E+04
609 PBMC 36 Months	87785	87785	54172	33066	61.71%	99.38%	4.88E+04
609 PBMC 48 Months	379459	379457	132841	233708	35.01%	96.60%	9.48E+03
609 T-Cells	301746	301745	108549	183399	35.97%	96.75%	8.40E+03

S15: Gene Therapy Patient Variants in the Endogenous *ADA* gene locus

	607				609			
**Sample**	Drug Product	PBMCs (18 Months)	PBMCs (48 Months)	T-Cells (24 Months)	Drug Product	PBMCs (36 Months)	PBMCs (48 Months)	T-Cells (24 Months)
**Total Mutations**	188	147	190	167	183	151	270	233
**Purine to Purine**								
A to G	1	3	2	1	0	2	7	6
G to A	175	128	158	133	172	133	220	191
**Pyrimidine to Pyrimidine**								
C to T	6	6	18	16	3	8	21	16
T to C	0	2	1	3	2	1	6	5
**Purine to Pyrimidine**								
A to C	0	0	1	0	0	0	1	3
A to T	0	0	0	0	0	1	0	1
G to C	0	0	0	0	0	0	1	0
G to T	0	0	0	2	0	0	0	1
**Pyrimidine To Purine**								
C to A	1	1	1	3	2	3	5	3
C to G	0	0	0	0	0	0	0	0
T to A	0	1	2	3	0	0	0	1
T to G	0	0	0	0	1	0	2	1
**Deletion/Insertion**	3	4	3	5	3	1	3	1
**Sample**	2	2	4	1	0	2	4	4

Table S16: Frequencies of Amino Acid Mutation Type Over Time

	607 Drug Product	607 PBMC 18 Months	607 PBMC 48 Months	607 T Cells	609 Drug Product	609 PBMC 36 Months	609 PBMC 48 Months	609 T Cells
Frequency Synonymous	42.9%	43.36%	46.7%	42.86%	43.97%	48.48%	44.44%	48.31%
Frequency Missense	51.7%	51.33%	48.0%	50.71%	51.77%	45.45%	50.24%	47.19%
Frequency Nonsense	4.08%	4.42%	3.33%	5.0%	4.26%	4.55%	3.38%	2.81%
Frequency Complex	1.32%	0.89%	1.97%	1.43%	0.00%	1.52%	1.94%	1.69%

Table S17: Sequencing Statistics of HEK293T RNA, Viral RNA, and DNA from ADA IDLV and Lentivirus transduced CD34+ cells

Sample	Read Total	Total Primary Alignments	Total Alignments to ADA or GFP Transgene	Total Alignments to Endogenous ADA or IL2	%On-Target Alignment (transgene)	%On-Target Alignment (Trans +Endogenous)	Transgene Mean Coverage
Sequencing Statistics Pre-Error Correction, Read Collapsing, and Filtering							
293T RNA_1	202741852	189772012	22001815	3976	11.59%	11.60%	5.42E+05
293T RNA_2	194045248	186560643	16060421	3426	8.61%	8.61%	3.82E+05
Viral RNA_1	214716000	158274737	126956694	7516	80.21%	80.22%	9.74E+05
Viral RNA_2	208914934	169520217	61756583	4266	36.43%	36.43%	9.71E+05
IDLV 1	418633474	415458708	142550507	7001243	34.31%	36.00%	9.73E+05
IDLV 2	374235964	371466189	134110815	58620242	36.10%	51.88%	9.74E+05
Lenti 1	210070248	208689292	48190546	40192894	23.09%	42.35%	9.73E+05
Lenti 2	250689140	249070093	58621672	49644759	23.54%	43.47%	9.75E+05
Sequencing Statistics Post-Error Correction, Read Collapsing, and Filtering							
293T RNA 1	3608968	3570991	1066117	70	29.85%	29.86%	2.41E+04
293T RNA 2	2415814	2391234	658787	54	27.55%	27.55%	1.31E+04
Viral RNA 1	9640516	9637548	9270705	4	96.19%	96.19%	2.81E+05
Viral RNA 2	4720708	4718597	4372223	0	92.66%	92.66%	1.18E+05
IDLV 1	683304	683273	395094	264725	57.82%	96.57%	1.16+04
IDLV 2	769346	769310	470234	274945	61.12%	96.86%	1.39E+04
Lenti 1	391092	391007	172851	200745	44.21%	95.55%	4.91E+03
Lenti 1	393304	393273	172783	201515	43.93%	95.18%	4.94E+03
